# Assessment of causal associations between handgrip strength and cardiovascular diseases: A two sample mendelian randomization study

**DOI:** 10.3389/fcvm.2022.930077

**Published:** 2022-08-04

**Authors:** Chengui Zhuo, Jianqiang Zhao, Qiqi Wang, Zujin Lin, Haipeng Cai, Huili Pan, Lei Chen, Xiangyu Jin, Hong Jin, Longwei Xu, Xiyan Tao

**Affiliations:** ^1^Department of Cardiology, Taizhou Central Hospital (Taizhou University Hospital), Taizhou, China; ^2^Department of Cardiology, The Fourth Affiliated Hospital of Zhejiang University School of Medicine, Yiwu, China; ^3^Department of Cardiology and Atrial Fibrillation Center, The First Affiliated Hospital, College of Medicine, Zhejiang University, Hangzhou, China; ^4^Department of Cardiology, The First Affiliated Hospital of Zhengzhou University, Zhengzhou, China

**Keywords:** handgrip strength, cardiovascular diseases, coronary artery disease, causal association, Mendelian randomization

## Abstract

**Background:**

Several observational studies have identified that handgrip strength was inversely associated with cardiovascular diseases (CVDs). Nevertheless, causality remains controversial. We conducted Mendelian randomization (MR) analysis to examine whether handgrip strength and risk of CVDs are causally associated.

**Methods:**

We identified 160 independent single nucleotide polymorphisms (SNPs) for right-hand grip strength and 136 independent SNPs for left-hand grip strength at the genome-wide significant threshold (*P* < 5 × 10^−8^) from UK Biobank participants and evaluated these in relation to risk of CVDs. MR estimates was calculated using the inverse-variance weighted (IVW) method and multiple sensitivity analysis was further conducted.

**Results:**

Genetical liability to handgrip strength was significantly associated with coronary artery disease (CAD) and myocardial infarction (MI), but not stroke, hypertension, or heart failure. Additionally, there was significant association between right-hand grip strength and atrial fibrillation (OR, 0.967; 95% CI, 0.950–0.984; *p* = 0.000222), however, suggestive significance was found between left-hand grip strength and atrial fibrillation (OR, 0.977; 95% CI, 0.957–0.998; *p* = 0.033). Results were similar in several sensitivity analysis.

**Conclusion:**

Our study provides support at the genetic level that handgrip strength is negatively associated with the risk of CAD, MI, and atrial fibrillation. Specific handgrip strength interventions on CVDs warrant exploration as potential CVDs prevention measures.

## Introduction

Cardiovascular diseases (CVDs) remain one of the leading causes of death globally, accounting for over 30% of all deaths, and place a heavy burden on health systems (1). The global burden of CVDs underscores the importance of exploring more effective prevention and treatment strategies. Traditional risk factors, including smoking (2), type 2 diabetes (3), body mass index (4), and lipid profile (5), have been found to aid the better management of CVDs. Besides, epidemiological studies have further identified the inverse association of handgrip strength with CVDs (6, 7).

As the most objective and simplest indicator of muscle strength (8), the relationship between handgrip strength and CVDs has been under the spotlight in recent years. A meta-analysis of 42 studies with 3,002,203 patients showed a negative linear association between handgrip strength and CVDs: The lower the handgrip strength, the higher the prevalence of CVDs (7). The hazard ratios and 95% confidence intervals (HRs and 95% CIs) with the per-5-kg decrease in handgrip strength was 1.21 (95% CIs, 1.14–1.29) for CVDs (7). However, most of the evidence for the meta-analysis comes from observational studies, which are inconclusive in identifying the causality because of the possibility of residual confounding and reverse causation.

With regard to the causal relationship, Mendelian Randomization (MR) is an increasingly applied approach that can use genetic variations from recent genome-wide association studies (GWASs) as instrumental variables (IVs) to clarify the causality between exposure and outcomes, and diminish potential confounding factors in observational studies (9, 10). Recently, a two-sample MR (TSMR) study showed that increased handgrip strength was causally related to a lower risk of CAD (11). However, the results of this TSMR study were still limited as only two single-nucleotide polymorphisms (SNPs) were selected as IVs and only the relationship between handgrip strength and CAD was explored. Therefore, in this study, we conduct the TSMR approach to examine the potential causality between handgrip strength and the risk of CVDs, including coronary artery disease (CAD), myocardial infarction (MI), atrial fibrillation (AF), heart failure (HF), ischemic stroke (IS) and its subtypes. And multiple complementary analysis also have been conducted to test the robustness of the results.

## Methods

### Study design

The TSMR analysis diagram is shown in Supplementary Figure 1. In short, the genetic variations used as IVs must follow three key assumptions: First, the genetic variants are strongly associated with handgrip strength (each genetic variant for handgrip strength reached genome-wide significance [*P* < 5 × 10^−8^], and the threshold of F statistic); Second, the genetic variants shouldn't be associated with any confounders; Third, the genetic variants effect the outcome only via the handgrip strength(no horizontal pleiotropy). All summary statistics presented in this study were derived from published GWAS on handgrip strength and CVDs (Supplementary Table 1).

### Data sources for handgrip strength and selection of IVs

The summary statistic for handgrip strength was derived from a recently released GWAS of the UK Biobank, which included ~360,000 participants from Europe (12). Briefly, this GWAS examined two handgrip strength phenotypes including right-hand grip strength (*n* = 359,729) and left-hand grip strength (*n* = 359,704). In the UK Biobank, handgrip strength was measured using a calibrated Jamar J00105 hydraulic hand dynamometer adjusted for hand size in five half-inch increments (13). We adopted absolute rather than relative handgrip strength as a marker, because absolute handgrip strength may be more correlated with physical capability than relative handgrip strength (8).

In order to meet the first assumption of MR analysis, this study selected 160 independent single-nucleotide polymorphisms (SNPs) associated with “right-hand grip strength” and 136 independent SNPs associated with “left-hand grip strength” at a genome-wide significance level (*P* < 5 × 10^−8^), using the PLINK clumping algorithm (r^2^ threshold = 0.001 and window size = 10 Mb) from the GWAS mentioned above. While SNPs for the handgrip strength phenotypes were unavailable in the outcome GWAS, proxy SNPs (linkage disequilibrium r^2^ > 0.8) were identified *via* an online website, available at https://ldlink.nci.nih.gov/. F statistics were generated to assess the strength of selected SNPs using the following formula: $F=\frac{R^{2}\left(N-2\right)}{\left(1-R^{2}\right)}$.

Where, R^2^ is the percentage of the variability in handgrip strength explained by the selected SNPs and N represents the sample size of the GWAS (14). An F-statistic >10 indicates a low risk of weak instrument bias in MR analysis (14).

### Data sources for CVDs

GWAS summary statistics for CVDs were extracted from: the CardiogramplusC4D consortium for CAD (60,801 cases and 123,504 controls) and MI (43,676 cases and 128,197 controls) (15); the HERMES Consortium for HF (47,309 cases and 930, 014 controls) (16); the HUNT, deCODE, the MGI, DiscovEHR, UK Biobank, and the AFGen Consortium for AF (65,446 cases and 522,744 controls) (17); Liu et al. for hypertension (146,562 individuals) (18), and the MEGASTROKE consortium for ischemic stroke (IS) (34 217 cases and 404 630 controls) (19). In line with the Trial of Org 10172 in Acute Stroke Treatment criteria, ischemic stroke was further categorized as large artery stroke (LAS), small vessel stroke (SVS), and cardioembolic stroke (CS) cases (20). Details of the datasets included in the analysis were shown in Supplementary Table 1.

### Statistical analysis

A TSMR method was conducted in this study. After extraction of data and harmonization of the effect alleles across GWASs, the MR estimates of the effect of handgrip strength on CVDs were calculated using the Wald estimates (21). The Delta method was used to account for possible measurement errors in the estimation of the causal association between handgrip strength and CVDs (21, 22). The fixed-effects inverse variance-weighted (IVW) method was adopted to evaluate the final effect estimate. Scatter plots of the MR effects estimated by each method were also provided.

Pleiotropy of SNPs in the IVW analysis may impact causal estimates and bias the results. In this study, we calculated the Cochran's Q to test the heterogeneity caused by different SNPs in the fixed-effects IVW. Cochran's Q *P*-value < 0.05 indicated the presence of heterogeneity, consequently, of horizontal pleiotropy (23). In cases with potential horizontal pleiotropy, the random-effects IVW method would be used. MR-Egger intercept test was conducted to detect potential directional pleiotropy, with an intercept *P*-value < 0.05 indicating significant pleiotropic bias (24).

Additionally, we also performed several sensitivity analysis to further ensure the robustness of our results, including the weighted median method, simple median method, MR-Egger regression method (24), MR pleiotropy residual sum, and outlier (MR-PRESSO) method (25), and leave-one-SNP-out method. In addition, $I_{\text{GX}}^{2}$ was calculated to test the potential weak IVs bias in the MR-Egger regression method. An $I_{\text{GX}}^{2}$ >95% means low risk of bias (26). MR-PRESSO could identify IVs which are likely to show pleiotropic effects (outlier IVs) and provided estimates after removing the outlier IVs (25). To rule out the IVs associated with any confounders that may affect handgrip strength and CVDs, we also searched each selected SNP and its proxies in Phenoscanner (27) and the GWAS catalog (28) for previously identified associations (*p*-value < 5 × 10^−6^) with relevant confounders or CVDs. In this study, smoking, drinking, body mass index (BMI), hypertension, diabetes, and lipid profile were regarded as confounders. We repeated the MR analysis mentioned above after removing the SNPs associated with relevant confounders or CVDs.

A two-sided *p*-value < 0.05 was set as suggestive significance, and due to the multiple comparisons, we further applied a Bonferroni corrected threshold for statistical significance (0.05/2^*^9 = 0.0028). All MR analysis were conducted using R software (version 3.5.4; www.r-project.org) with the R packages “Mendelian Randomization”, “MRPRESSO” and “TwosampleMR”.

## Result

Supplementary Tables 2, 3 shows the characteristics of all correlated SNPs for handgrip strength. In total, we extracted 160 and 136 independent SNPs that reached genome-wide significance from right-hand grip strength and left-hand grip strength, respectively. Most SNPs were available in the GWAS of CVDs except for HF and atrial fibrillation (rs57884925 was not available for HF and AF). Thus, we found the proxy-SNP (rs7034200) to replace it. Among all selected SNPs, the F statistics were higher than 10 and ranged from 30 to 159. In the PhenoScanner and GWAS catalog, we identified 33 (rs7034200 was associated with diabetes while rs57884925 was not) and 27 selected SNPs that were considered to be associated with confounders or CVDs for right-hand grip strength and left-hand grip strength, respectively (Supplementary Tables 4, 5).

Cochran's Q test had indicated the presence of significant heterogeneity in some MR analysis (*P*-value < 0.05, Supplementary Table 6), consequently, for these models random-effects IVW methods were conducted. According to the IVW analysis, both genetically predicted right- and left-hand grip strength were significantly negatively associated with CAD or MI (Tables 1, 2). There was significant association between right-hand grip strength and AF (OR, 0.967; 95% CI, 0.950–0.984; *p* = 0.000222), however, suggestive evidence was found between left-hand grip strength and AF (OR,0.977; 95% CI,0.957-0.998; *p* = 0.033). In sensitivity analysis, the causal association of handgrip strength with CAD, MI, and AF was confirmed using the weighted median, simple median, MR-PRESSO, MR-Egger regression (Supplementary Tables 7, 8), and leave-one-SNP-out method (Supplementary Figures 2–7). $I_{\text{GX}}^{2}$ for right- and left-hand grip strength was higher than 0.95, indicating a low chance of weak IVs bias in MR-Egger regression (Supplementary Tables 7, 8). Importantly, the MR-PRESSO method had detected some outliers, but the results were similar after excluding the outliers (Tables 1, 2; Supplementary Table 9).

**Table 1:** Mendelian randomization estimates between right handgrip strength and cardiovascular diseases.

**Outcomes**	**SNP selection**	**No. of SNPs**	**IVW**		**Weighted median**		**Simple median**		**MR-Egger**		**MR-PRESSO**^**†**^ **(outlier-corrected)**	
			**OR (95% CI)**	***p-*value**	**OR (95% CI)**	***p-*value**	**OR (95% CI)**	***p-*value**	**OR (95% CI)**	***p-*value**	**OR (95% CI)**	***p-*value**
CAD	All	160	0.956 (0.938–0.974)	2.16E-06*	0.953 (0.932–0.975)	2.87E-05*	0.957 (0.936–0.979)	1.11E-04*	0.888 (0.816–0.966)	0.006^#^	0.962 (0.945–0.978)	1.76E-05*
	Remove	128	0.957 (0.939–0.976)	8.08E-06*	0.952 (0.929–0.976)	8.66E-05*	0.957 (0.934–0.98)	3.65E-04*	0.901 (0.828–0.981)	0.016^#^	NA	NA
MI	All	160	0.957 (0.938–0.977)	3.30E-05*	0.959 (0.935–0.983)	7.71E-04*	0.966 (0.943–0.990)	0.006^#^	0.866 (0.790–0.949)	0.002*	0.957 (0.938–0.976)	1.89E-05*
	Remove	128	0.958 (0.938–0.979)	8.41E-05*	0.956 (0.931–0.982)	0.001*	0.966 (0.941–0.992)	0.011^#^	0.880 (0.802–0.965)	0.007^#^	NA	NA
AF	All	160	0.967 (0.950–0.984)	2.22E-04*	0.969 (0.953–0.986)	2.61E-04*	0.979 (0.963–0.996)	0.015^#^	0.913 (0.846–0.986)	0.020^#^	0.967 (0.952–0.982)	3.60E-05*
	Remove	127	0.964 (0.947–0.981)	5.26E-05*	0.972 (0.953–0.990)	0.003^#^	0.979 (0.961–0.998)	0.030^#^	0.942 (0.874–1.016)	0.120	0.963 (0.947–0.98)	4.18E-05*
HF	All	160	1.003 (0.989–1.018)	0.667	1.006 (0.988–1.024)	0.505	1.002 (0.984–1.020)	0.846	0.992 (0.932–1.056)	0.796	NA	NA
	Remove	127	1.002 (0.987–1.017)	0.836	1.006 (0.986–1.027)	0.550	1.001 (0.981–1.021)	0.925	0.986 (0.926–1.051)	0.667	NA	NA
Hypertension	All	160	0.986 (0.957–1.015)	0.333	1.006 (0.967–1.046)	0.757	1.008 (0.970–1.048)	0.678	0.983 (0.857–1.128)	0.806	NA	NA
	Remove	128	0.979 (0.947–1.011)	0.197	1.007 (0.964–1.053)	0.747	1.006 (0.964–1.050)	0.787	0.964 (0.833–1.115)	0.621	NA	NA
IS	All	160	0.995 (0.979–1.012)	0.592	1.002 (0.979–1.026)	0.873	1.003 (0.980–1.026)	0.817	0.959 (0.890–1.034)	0.276	NA	NA
	Remove	128	0.996 (0.977–1.015)	0.664	1.003 (0.977–1.029)	0.831	1.011 (0.987–1.037)	0.370	0.962 (0.888–1.043)	0.350	NA	NA
CS	All	160	0.999 (0.969–1.030)	0.954	0.993 (0.952–1.036)	0.753	0.985 (0.944–1.028)	0.496	0.990 (0.862–1.137)	0.886	NA	NA
	Remove	128	0.988 (0.954–1.023)	0.500	0.988 (0.942–1.037)	0.630	0.985 (0.939–1.033)	0.527	0.977 (0.840–1.137)	0.766	NA	NA
LAS	All	160	0.959 (0.922–0.999)	0.042^#^	0.957 (0.905–1.013)	0.133	0.966 (0.914–1.021)	0.223	0.859 (0.718–1.028)	0.097	NA	NA
	Remove	128	0.949 (0.910–0.99)	0.015^#^	0.961 (0.902–1.023)	0.211	0.970 (0.912–1.03)	0.320	0.850 (0.708–1.021)	0.082	NA	NA
SVS	All	160	1.016 (0.981–1.052)	0.369	1.198 (1.137–1.263)	1.10E-11*	1.213 (1.151–1.277)	3.02E-13*	1.217 (1.044–1.420)	0.012^#^	NA	NA
	Remove	128	1.019 (0.980–1.060)	0.340	1.206 (1.136–1.280)	9.28E-10*	1.233 (1.161–1.308)	5.84E-12*	1.257 (1.063–1.485)	0.007^#^	NA	NA

*CAD, coronary artery disease; MI, myocardial infarction; AF, atrial fibrillation; HF, heart failure; IS, ischemic stroke; CS, cardioembolic stroke; LAS, large artery stroke; SVS, small vessel stroke; OR, odds ratio; CI, confidence intervals, IVW, inverse-variance-weighted method; MR-PRESSO, MR pleiotropy residual sum and outlier method; SNPs, single-nucleotide polymorphisms*.

*p < 0.0028.

*^#^p < 0.05*.

*^†^Details of outliers were displayed in Supplementary Table 9*.

**Table 2:** Mendelian randomization estimates between left handgrip strength and cardiovascular diseases.

**Outcomes**	**SNP selection**	**No. of SNPs**	**IVW**		**Weighted median**		**Simple median**		**MR-Egger**		**MR-PRESSO**^**†**^ **(outlier-corrected)**	
			**OR (95% CI)**	***p-*value**	**OR (95% CI)**	***p-*value**	**OR (95% CI)**	***p-*value**	**OR (95% CI)**	***p-*value**	**OR (95% CI)**	***p-*value**
CAD	All	136	0.958 (0.939–0.977)	2.11E-05*	0.960 (0.938–0.983)	6.60E-04*	0.960 (0.938–0.982)	5.27E-04*	0.948 (0.858–1.047)	0.292	0.958 (0.94–0.977)	2.21E-05*
	Remove	109	0.962 (0.942–0.983)	5.12E-04*	0.970 (0.945–0.996)	0.022^#^	0.969 (0.944–0.995)	0.019^#^	0.957 (0.859–1.067)	0.429	NA	NA
MI	All	136	0.960 (0.940–0.981)	1.90E-04*	0.964 (0.939–0.989)	0.005^#^	0.959 (0.935–0.984)	0.002*	0.944 (0.849–1.050)	0.287	0.958 (0.938–0.978)	7.84E-05
	Remove	109	0.963 (0.942–0.986)	0.001*	0.969 (0.941–0.997)	0.029^#^	0.967 (0.940–0.995)	0.022^#^	0.974 (0.869–1.092)	0.655	NA	NA
AF	All	136	0.977 (0.957–0.998)	0.033^#^	0.972 (0.955–0.989)	0.001*	0.973 (0.956–0.99)	0.002*	0.895 (0.808–0.992)	0.034^#^	0.979 (0.964–0.994)	6.49E-03^#^
	Remove	109	0.978 (0.956–1.000)	0.045^#^	0.974 (0.955–0.993)	0.008^#^	0.974 (0.955–0.994)	0.009^#^	0.932 (0.835–1.040)	0.208	0.980 (0.963–0.997)	0.024^#^
HF	All	136	1.004 (0.990–1.018)	0.571	1.003 (0.984–1.022)	0.754	1.003 (0.984–1.022)	0.757	1.013 (0.946–1.085)	0.708	NA	NA
	Remove	109	1.003 (0.988–1.019)	0.698	1.001 (0.98–1.023)	0.91	1.001 (0.980–1.023)	0.933	1.027 (0.953–1.106)	0.483	NA	NA
Hypertension	All	136	0.965 (0.935–0.997)	0.032^#^	0.957 (0.919–0.996)	0.033^#^	0.967 (0.928–1.007)	0.1	1.043 (0.891–1.221)	0.602	NA	NA
	Remove	109	0.960 (0.927–0.995)	0.025^#^	0.964 (0.921–1.010)	0.125	0.978 (0.934–1.024)	0.35	1.046 (0.880–1.242)	0.613	NA	NA
IS	All	136	0.996 (0.979–1.013)	0.62	0.991 (0.967–1.015)	0.465	0.990 (0.966–1.015)	0.436	1.01 (0.927–1.099)	0.822	NA	NA
	Remove	109	0.994 (0.976–1.012)	0.506	0.991 (0.965–1.018)	0.522	0.990 (0.963–1.017)	0.473	1.014 (0.924–1.112)	0.77	NA	NA
CS	All	136	1.015 (0.983–1.047)	0.36	1.011 (0.966–1.058)	0.642	1.010 (0.965–1.057)	0.664	1.102 (0.936–1.298)	0.242	NA	NA
	Remove	109	1.01 (0.975–1.047)	0.568	1.008 (0.960–1.059)	0.742	1.008 (0.960–1.059)	0.742	1.051 (0.875–1.263)	0.594	NA	NA
LAS	All	136	0.984 (0.945–1.026)	0.453	1.001 (0.943–1.063)	0.969	0.998 (0.940–1.06)	0.955	0.95 (0.767–1.176)	0.636	NA	NA
	Remove	109	0.979 (0.935–1.025)	0.371	0.997 (0.934–1.065)	0.938	0.996 (0.932–1.064)	0.901	0.979 (0.773–1.241)	0.862	NA	NA
SVS	All	136	0.976 (0.938–1.016)	0.233	0.952 (0.901–1.007)	0.085	0.946 (0.895–1.000)	0.0498^#^	1.013 (0.827–1.241)	0.901	NA	NA
	Remove	109	0.970 (0.926–1.016)	0.199	0.938 (0.880–1.000)	0.049^#^	0.936 (0.878–0.998)	0.044^#^	1.014 (0.798–1.288)	0.91	NA	NA

*CAD, coronary artery disease; MI, myocardial infarction; AF, atrial fibrillation; HF, heart failure; IS, ischemic stroke; CS, cardioembolic stroke; LAS, large artery stroke; SVS, small vessel stroke; OR, odds ratio; CI, confidence intervals, IVW, inverse-variance-weighted method; MR-PRESSO, MR pleiotropy residual sum and outlier method; SNPs, single-nucleotide polymorphisms*.

*p < 0.0028.

*^#^p < 0.05*.

*^†^Details of outliers were displayed in Supplementary Table 9*.

On the flip side, we found that right-hand grip strength and left-hand grip strength were suggestively inversely associated with hypertension and large artery stroke, respectively, but these findings were inconsistent in sensitivity analysis (Tables 1, 2). For other CVDs outcomes (HF, IS, CS, SVS), no significant association was further identified. Directional pleiotropy was only found in the association of right-hand grip strength with SVS (Supplementary Tables 7, 8), which may impact the result. To get more robust results, we further removed the SNPs associated with any confounders or CVDs and the causal estimates were consistent (Tables 1, 2). Scatter plots depicting the MR effect evaluated by each method were also displayed in Supplementary Figures 8–11.

## Discussion

In this study, we explored the causal associations between handgrip strength and CVDs by using TSMR analysis. We confirmed that greater handgrip strength was significantly causally associated with the lower risk of CAD and MI. Additionally, there was a significant association between right-hand grip strength and AF, while suggestive significance was detected between left-hand grip strength and AF. Besides, no significant associations of handgrip strength with HF, hypertension, IS, CS, LAS, and SVS were found.

The observational studies that suggested handgrip strength may be associated with CAD and MI have inspired researchers to search for more evidence to demonstrate the causal association (8, 29). TSMR analysis has been applied in previous studies to investigate the causal association, but the results were inconsistent (11, 30). Xu et al. used 2 SNPs as genetic variants for handgrip strength and reported inverse causal associations of handgrip strength with the risk of CAD or MI (11). In contrast, Willems et al. identified 16 SNPs associated with handgrip strength and did not detect any apparent association between handgrip strength and cardiovascular events (CAD or MI) (30). The discrepancy might be attributed to the limited number of SNPs (2 and 16 SNPs for two studies), pleiotropic bias, different data sources, and statistical analysis. Recently, Liu et al. also identified the causal association between handgrip strength and CAD by using TSMR (31). However, the results of Liu et al. were still restricted as just 95 and 81 SNPs were identified as IVs for right- and left- handgrip strength, and only the association between handgrip strength and CAD was evaluated (31). In this TSMR study, we extracted a total of 160 and 136 SNPs as genetic variants for right-hand grip strength and left-hand grip strength, and indicated a negative association between handgrip strength and CAD or MI. These results remained robust after removing potential pleiotropic IVs through several sensitivity analysis. As handgrip strength was positively correlated with muscle mass (32), which is the primary site of glucose disposal (33), a potential mechanism for the association between handgrip strength and CAD or MI may be related to increased insulin action and decreased blood glucose in people with higher handgrip strength.

Our findings were consistent with a previous observational study showing that handgrip strength was negatively association with AF (34). The results of this study indicated that the HRs were 0.73 (95%CI, 0.61–0.86) for AF per one standard deviation increase in handgrip strength. Additionally, another cohort study with 1.1 million participants also confirmed that handgrip strength was significantly related to the risk of arrhythmia. After a median follow-up of 26.3 years, the HRs were 0.92 (95%CI, 0.61–0.86) for arrhythmia compared with low handgrip strength. In this TSMR study, we further divided handgrip strength into right- and left-hand grip strength, and there was suggestive evidence for the association of left-hand grip strength with AF while a significant causal association was identified between right-hand grip strength and AF. One possible reason for the difference is that approximately 89% of people in the UK biobank had right-hand preference (35), which may lead to selection bias and influence the results (36).

The causal association between handgrip strength and stroke remains inconclusive till now. A prospective study including 12,237 participants showed that handgrip weakness had 89.3% higher risk of stroke (37). However, Andersen et al. revealed that there was no significant association of stroke risk with higher muscle strength (HRs 1.01; 95%CI 0.94–1.10) (38). To our knowledge, this may be the first TSMR study to assess the causal association of handgrip strength with stroke. We observed that right- and left-hand grip strength was not causally associated with stroke and its subtypes. Similarly, the role of handgrip strength in stroke has not been highlighted in existing guidelines (39). Additionally, we further revealed that handgrip strength was not associated with the risk of HF and hypertension which is in line with previous studies (40, 41).

Our study has several evident strengths. Firstly, this was the first TSMR study to evaluate the causal associations of handgrip strength with AF, HF, IS, and its subtypes by using the recently published GWAS. Secondly, various complementary analysis were applied to address pleiotropic bias and ensure the robustness of our results. Thirdly, we repeated the analysis after excluding the IVs associated with any confounders or CVDs and the result was consistent.

Besides, several potential limitations were also worth acknowledging. Firstly, while no apparent pleiotropy was detected for the IVs used, the possibility of residual pleiotropy still cannot be completely ruled out. There may be other undiscovered causal pathways of handgrip strength with CVDs. Second, SNPs associated with handgrip strength were selected from the GWAS of UK Biobank, which consists of participants aged between 40 to 70 years from Europe. Furthermore, we do not have the demographic information which restricts the generalizability of our results. Thus, further studies are warranted to confirm our findings on other populations. Third, though handgrip strength is an objective and common marker of muscular strength, it mainly represents upper body strength. Fourth, as the causal association was evaluated using MR based on the genetic information of each trait, the result should be interpreted with caution (42), with the understanding that the development of handgrip strength and CVDs were multifactorial and involved interactions among plenty of psycho-social-environmental factors (43). Finally, some samples in the GWAS of AF and HF have also been included in the UK Biobank, which may introduce bias. However, this bias would likely be minimal due to the limited overlap in the samples between handgrip strength and CVDs (22% for AF and 6% for HF).

## Conclusion

To sum up, our study provides genetic evidence supporting a causal association between handgrip strength on CAD, MI, and AF, but not stroke, hypertension, or heart failure. Given the significance of these associations, specific handgrip strength interventions could be further investigated as potential CVDs prevention measures.

## Data availability statement

The datasets presented in this study can be found in online repositories. The names of the repository/repositories and accession number(s) can be found in the article/Supplementary material.

## Ethics statement

Ethical review and approval was not required for this study in accordance with the local legislation and institutional requirements.

## Author contributions

XT, CZ, and JZ conceived and designed the study. ZL, CZ, and QW drafted the paper. HP, LC, and HJ collected the data. CZ, XT, XJ, and HC analyzed and interpreted the data. LX consulted literatures and helped the language editing. All authors contributed to the article and approved the submitted version.

## Funding

This study was supported by the grant from the Department of Science and Technology, Zhejiang Province (Grant No. LGF19H020011).

## Conflict of interest

The authors declare that the research was conducted in the absence of any commercial or financial relationships that could be construed as a potential conflict of interest.

## Publisher's note

All claims expressed in this article are solely those of the authors and do not necessarily represent those of their affiliated organizations, or those of the publisher, the editors and the reviewers. Any product that may be evaluated in this article, or claim that may be made by its manufacturer, is not guaranteed or endorsed by the publisher.
