# Supplementary material for: Assessment of causal associations between handgrip strength and cardiovascular diseases: A two sample mendelian randomization study
Source: Front Cardiovasc Med. 2022 Aug 4;9:930077. doi: 10.3389/fcvm.2022.930077 (PMC9386423; doi:10.3389/fcvm.2022.930077)
Supplement: Supplementary file 1 [file Data_Sheet_1.docx]

Supplementary Material

[Supplementary Table 1. Details of the studies and datasets included in the analyses. 1](#_Toc7778)

[Supplementary Table 2.Genome-wide significant SNPs for right handgrip strength. 2](#_Toc13081)

[Supplementary Table 3.Genome-wide significant SNPs for left handgrip strength. 6](#_Toc25827)

[Supplemental Table 4. Evidence of association (p<5*10-6) of the SNPs used as genetic variants for Mendelian randomization analyses of right hangrip strength with confounders or CVDs in the PhenoScanner and the GWAS catalog. 10](#_Toc16994)

[Supplemental Table 5. Evidence of association (p<5*10-6) of the SNPs used as genetic variants for Mendelian randomization analyses of left hangrip strength with confounders or CVDs in the PhenoScanner and the GWAS catalog. 14](#_Toc18751)

[Supplemental Table 6. Heterogeneity for the Mendelian randomization analysis. 18](#_Toc11983)

[Supplemental Table 7. MR-Egger pleiotropy test for all SNPS. 19](#_Toc18200)

[Supplemental Table 8. MR-Egger pleiotropy test after removing SNPs associated with confounders or CVDs. 20](#_Toc15615)

[Supplemental Table 9.SNPs excluded from the outlier corrected MR-PRESSO analyses between handgrip strength and cardiovascular diseases. 21](#_Toc4602)

[Supplemental Figure 1.The diagram of two-sample MR analysis. 23](#_Toc22056)

[Supplemental Figure 2.MR Leave one out analyses for right handgrip strength on coronary artery disease. 24](#_Toc19118)

[Supplemental Figure 3.MR Leave one out analyses for right handgrip strength on myocardial infarction. 26](#_Toc19100)

[Supplemental Figure 4.MR Leave one out analyses for right handgrip strength on atrial fibrillation. 28](#_Toc4165)

[Supplemental Figure 5.MR Leave one out analyses for left handgrip strength on coronary artery disease. 30](#_Toc5407)

[Supplemental Figure 6.MR Leave one out analyses for left handgrip strength on 32](#_Toc21123)

[myocardial infarction. 32](#_Toc3633)

[Supplemental Figure 7.MR Leave one out analyses for left handgrip strength on atrial fibrillation. 34](#_Toc14474)

[Supplemental Figure 8.A scatter plot for the causal association of right handgrip strength with outcomes. 36](#_Toc13955)

[Supplemental Figure 9.A scatter plot for the causal association of right handgrip strength with outcomes after removing the SNPs associated with confounders or CVDs. 37](#_Toc17912)

[Supplemental Figure 10.A scatter plot for the causal association of left handgrip strength with outcomes. 38](#_Toc14890)

[Supplemental Figure 11.A scatter plot for the causal association of right handgrip strength with outcomes after removing the SNPs associated with confounders or CVDs. 39](#_Toc31186)

# Supplementary Table 1. Details of the studies and datasets included in the analyses.

| **GWAS** | **Phenotype** | **Participants** | **Ancestry** | **Use in this MR study** | **Adjustments*** |
| --- | --- | --- | --- | --- | --- |
| UKbiobank^10^ | Right handgrip strength | 359,729 individuals | European | Exposure | age, sex, sex×age and  sex×age2 |
|  | Left handgrip strength | 359,704  individuals |  |  |  |
| CARDIoGRAMplusC4D^13^ | Coronary artery disease | 60,801 cases  123,504 controls | Multi-ancestry  (77% European) | outcome | age, sex |
|  | Myocardial infarction | 43,676 cases  128,197 controls |  |  |  |
| HERMES Consortium^14^ | Heart failure | 47,309 cases  930,014 controls | European | outcome | age, sex, genotypin array |
| Nielsen et al,2018^15^ | Atrial fibrillation | 60,620 cases  970,216 controls | European | outcome | age, sex |
| MEGASTROKE^17^ | Any ischemic stroke | 34,217 cases  404,630 controls | European | outcome | age, sex |
|  | Large artery stroke | 4373 cases 146392 controls |  |  |  |
|  | Small vessel stroke | 5386 cases and 192662 controls |  |  |  |
|  | Cardioembolic stroke | 7193 cases and 204570 controls |  |  |  |
| Liu et al，2016^16^ | Hypertension | 146,562 individuals | Multi-ancestry  (82% European) | outcome | age, age^2^, sex, and body mass index |

*All GWAS studies have further adjusted for principal components.

# Supplementary Table 2.Genome-wide significant SNPs for right handgrip strength.

| SNP | Proxy SNP | r2 for proxy | CHR | Position | OA | EA | EAF | BETA | SE | P-value | N | R^2^ | F statistic | Genes |
| --- | --- | --- | --- | --- | --- | --- | --- | --- | --- | --- | --- | --- | --- | --- |
| rs10041126 | - | - | 5 | 52813119 | A | C | 0.603 | 0.109 | 0.018 | 3.63E-09 | 359729 | 0.0001 | 35 | AC116606.1 |
| rs10176878 | - | - | 2 | 59952274 | T | C | 0.192 | -0.124 | 0.023 | 4.90E-08 | 359729 | 0.00008 | 30 | AC007131.2 |
| rs10185503 | - | - | 2 | 218120475 | T | C | 0.252 | -0.113 | 0.021 | 3.91E-08 | 359729 | 0.00008 | 30 | RN7SKP43 |
| rs10273327 | - | - | 7 | 46248930 | C | G | 0.544 | 0.101 | 0.018 | 3.02E-08 | 359729 | 0.00009 | 31 | AC023669.1 |
| rs10278546 | - | - | 7 | 100516003 | A | C | 0.195 | 0.126 | 0.023 | 2.02E-08 | 359729 | 0.00009 | 31 | RPS29P15 |
| rs10445885 | - | - | 2 | 44116100 | C | T | 0.472 | 0.115 | 0.018 | 1.88E-10 | 359729 | 0.00011 | 41 | LRPPRC |
| rs1047437 | - | - | 5 | 122685727 | C | G | 0.168 | -0.130 | 0.024 | 4.24E-08 | 359729 | 0.00008 | 30 | CEP120 |
| rs10496731 | - | - | 2 | 135597628 | T | G | 0.363 | 0.126 | 0.019 | 1.17E-11 | 359729 | 0.00013 | 46 | ACMSD,CCNT2-AS1 |
| rs1065778 | - | - | 15 | 51520206 | T | C | 0.513 | 0.099 | 0.018 | 2.76E-08 | 359729 | 0.00009 | 31 | CYP19A1 |
| rs1076635 | - | - | 6 | 15516078 | A | G | 0.267 | -0.146 | 0.020 | 5.45E-13 | 359729 | 0.00014 | 52 | JARID2 |
| rs10788958 | - | - | 1 | 54040670 | C | G | 0.647 | 0.132 | 0.019 | 2.42E-12 | 359729 | 0.00014 | 49 | GLIS1 |
| rs10797937 | - | - | 1 | 184017131 | A | G | 0.349 | 0.102 | 0.019 | 4.06E-08 | 359729 | 0.00008 | 30 | TSEN15 |
| rs10851633 | - | - | 15 | 58323065 | C | T | 0.375 | -0.104 | 0.018 | 1.44E-08 | 359729 | 0.00009 | 32 | ALDH1A2 |
| rs10998287 | - | - | 10 | 70330892 | C | T | 0.396 | 0.103 | 0.018 | 1.30E-08 | 359729 | 0.00009 | 32 | TET1 |
| rs11067228 | - | - | 12 | 115094260 | A | G | 0.449 | -0.099 | 0.018 | 3.16E-08 | 359729 | 0.00009 | 31 | OSTF1P1, Y_RNA |
| rs11072542 | - | - | 15 | 75634599 | G | A | 0.755 | -0.113 | 0.021 | 4.28E-08 | 359729 | 0.00008 | 30 | NEIL1 |
| rs11121529 | - | - | 1 | 10271688 | C | G | 0.114 | -0.173 | 0.028 | 6.19E-10 | 359729 | 0.00011 | 38 | KIF1B |
| rs11125160 | - | - | 2 | 23891759 | G | A | 0.673 | 0.114 | 0.019 | 1.92E-09 | 359729 | 0.0001 | 36 | KLHL29 |
| rs11135316 | - | - | 5 | 163939367 | A | C | 0.277 | -0.111 | 0.020 | 2.35E-08 | 359729 | 0.00009 | 31 | CTC-340A15.2 |
| rs112570672 | - | - | 4 | 17914257 | G | A | 0.044 | -0.270 | 0.043 | 5.58E-10 | 359729 | 0.00011 | 38 | LCORL, KRT18P63 |
| rs112852122 | - | - | 20 | 47498117 | G | A | 0.160 | 0.139 | 0.025 | 1.60E-08 | 359729 | 0.00009 | 32 | PREX1,ARFGEF2,CSE1L |
| rs1138120 | - | - | 22 | 30229954 | T | C | 0.385 | -0.101 | 0.018 | 4.06E-08 | 359729 | 0.00008 | 30 | ASCC2 |
| rs113835839 | - | - | 6 | 13784625 | C | T | 0.248 | -0.113 | 0.021 | 3.83E-08 | 359729 | 0.00008 | 30 | MCUR1 |
| rs11546878 | - | - | 3 | 183976103 | C | T | 0.175 | -0.135 | 0.023 | 9.52E-09 | 359729 | 0.00009 | 33 | EEF1AKMT4-ECE2, EEF1AKMT4 |
| rs115771255 | - | - | 3 | 132119691 | A | T | 0.100 | 0.165 | 0.030 | 3.09E-08 | 359729 | 0.00009 | 31 | NIP7P2 |
| rs11584359 | - | - | 1 | 23293972 | C | T | 0.176 | -0.136 | 0.023 | 6.93E-09 | 359729 | 0.00009 | 34 | LACTBL1 |
| rs116222218 | - | - | 15 | 99196678 | C | T | 0.048 | -0.244 | 0.042 | 6.05E-09 | 359729 | 0.00009 | 34 | IGF1R |
| rs11659241 | - | - | 18 | 46588476 | C | T | 0.470 | 0.111 | 0.018 | 4.46E-10 | 359729 | 0.00011 | 39 | DYM |
| rs116725229 | - | - | 1 | 86236934 | T | G | 0.144 | 0.141 | 0.025 | 2.86E-08 | 359729 | 0.00009 | 31 | COL24A1 |
| rs11676702 | - | - | 2 | 68410587 | T | C | 0.080 | -0.210 | 0.033 | 1.59E-10 | 359729 | 0.00011 | 41 | PPP3R1 |
| rs116782923 | - | - | 5 | 102331465 | A | T | 0.054 | -0.217 | 0.040 | 4.29E-08 | 359729 | 0.00008 | 30 | PAM |
| rs116922558 | - | - | 9 | 118802375 | A | G | 0.040 | -0.276 | 0.046 | 1.72E-09 | 359729 | 0.0001 | 36 | RP11-787B4.2 |
| rs11810706 | - | - | 1 | 176001826 | C | T | 0.339 | 0.104 | 0.019 | 3.53E-08 | 359729 | 0.00008 | 30 | RFWD2 |
| rs11854314 | - | - | 15 | 74101300 | C | T | 0.112 | -0.194 | 0.029 | 1.11E-11 | 359729 | 0.00013 | 46 | C15orf59-AS1 |
| rs11857557 | - | - | 15 | 56793502 | G | A | 0.235 | -0.122 | 0.021 | 1.38E-08 | 359729 | 0.00009 | 32 | MNS1 |
| rs11887431 | - | - | 2 | 42267462 | C | T | 0.233 | 0.118 | 0.021 | 2.45E-08 | 359729 | 0.00009 | 31 | PKDCC |
| rs11949931 | - | - | 5 | 67784200 | T | C | 0.620 | -0.103 | 0.019 | 4.88E-08 | 359729 | 0.00008 | 30 | JMY |
| rs12099669 | - | - | 12 | 46783653 | G | A | 0.695 | 0.111 | 0.019 | 1.07E-08 | 359729 | 0.00009 | 33 | RP11-474P2.6 |
| rs12425282 | - | - | 12 | 15051689 | T | C | 0.201 | -0.140 | 0.022 | 3.12E-10 | 359729 | 0.00011 | 40 | C12orf60 |
| rs12740679 | - | - | 1 | 150262270 | C | G | 0.257 | 0.113 | 0.020 | 3.36E-08 | 359729 | 0.00008 | 30 | CIART, MRPS21 |
| rs12889267 | - | - | 14 | 21542766 | A | G | 0.168 | -0.136 | 0.024 | 1.04E-08 | 359729 | 0.00009 | 33 | ARHGEF40 |
| rs12928404 | - | - | 16 | 28847246 | T | C | 0.410 | -0.100 | 0.018 | 3.38E-08 | 359729 | 0.00008 | 30 | ATXN2L |
| rs12949046 | - | - | 17 | 63555147 | C | G | 0.564 | 0.100 | 0.018 | 2.37E-08 | 359729 | 0.00009 | 31 | AXIN2 |
| rs12991919 | - | - | 2 | 44995766 | G | A | 0.383 | -0.104 | 0.018 | 1.78E-08 | 359729 | 0.00009 | 32 | CAMKMT |
| rs13011633 | - | - | 2 | 179635919 | C | T | 0.035 | -0.301 | 0.048 | 4.56E-10 | 359729 | 0.00011 | 39 | TTN |
| rs13029742 | - | - | 2 | 220035118 | C | A | 0.304 | -0.115 | 0.019 | 3.00E-09 | 359729 | 0.0001 | 35 | CNPPD1, SLC23A3 |
| rs13105682 | - | - | 4 | 102702364 | T | G | 0.059 | -0.214 | 0.039 | 3.57E-08 | 359729 | 0.00008 | 30 | BANK1 |
| rs13298297 | - | - | 9 | 119264108 | G | A | 0.230 | 0.125 | 0.021 | 4.48E-09 | 359729 | 0.0001 | 34 | ASTN2 |
| rs13399936 | - | - | 2 | 48654126 | T | G | 0.298 | 0.108 | 0.020 | 4.16E-08 | 359729 | 0.00008 | 30 | RP11-191L17.1 |
| rs139603701 | - | - | 1 | 2904634 | A | G | 0.017 | -0.397 | 0.072 | 3.33E-08 | 359729 | 0.00008 | 31 | ACTRT2 |
| rs1418282 | - | - | 1 | 34800533 | A | G | 0.251 | -0.112 | 0.021 | 4.86E-08 | 359729 | 0.00008 | 30 | RP4-657M3.2 |
| rs1440152 | - | - | 3 | 98489915 | C | G | 0.445 | 0.110 | 0.018 | 9.71E-10 | 359729 | 0.0001 | 37 | ST3GAL6 |
| rs1480474 | - | - | 12 | 66326943 | A | G | 0.414 | -0.128 | 0.018 | 1.47E-12 | 359729 | 0.00014 | 50 | HMGA2 |
| rs1556660 | - | - | 10 | 130834551 | A | G | 0.590 | 0.139 | 0.018 | 2.42E-14 | 359729 | 0.00016 | 58 | RP11-442O18.1 |
| rs1558461 | - | - | 7 | 2121156 | G | A | 0.120 | -0.150 | 0.027 | 4.86E-08 | 359729 | 0.00008 | 30 | MAD1L1 |
| rs16910750 | - | - | 9 | 99084471 | G | C | 0.159 | 0.149 | 0.024 | 1.04E-09 | 359729 | 0.0001 | 37 | SLC35D2 |
| rs17008195 | - | - | 3 | 71129879 | C | T | 0.128 | -0.154 | 0.027 | 1.16E-08 | 359729 | 0.00009 | 33 | FOXP1 |
| rs17046434 | - | - | 2 | 24872463 | C | T | 0.074 | -0.196 | 0.034 | 8.77E-09 | 359729 | 0.00009 | 33 | NCOA1 |
| rs17098787 | - | - | 10 | 121130578 | G | A | 0.100 | 0.164 | 0.030 | 3.33E-08 | 359729 | 0.00008 | 31 | GRK5 |
| rs17368853 | - | - | 1 | 160169230 | G | A | 0.037 | -0.306 | 0.048 | 1.25E-10 | 359729 | 0.00012 | 41 | CASQ1 |
| rs17536644 | - | - | 7 | 120721025 | A | G | 0.440 | 0.100 | 0.018 | 2.79E-08 | 359729 | 0.00009 | 31 | CPED1 |
| rs17631394 | - | - | 17 | 61646000 | G | A | 0.263 | 0.117 | 0.020 | 9.31E-09 | 359729 | 0.00009 | 33 | DCAF7 |
| rs185490114 | - | - | 6 | 31035118 | G | A | 0.222 | -0.134 | 0.021 | 4.24E-10 | 359729 | 0.00011 | 39 | TBC1D22B |
| rs1883640 | - | - | 6 | 20529896 | T | C | 0.723 | 0.119 | 0.020 | 3.14E-09 | 359729 | 0.0001 | 35 | CDKAL1 |
| rs188412997 | - | - | 7 | 69101952 | C | T | 0.044 | -0.237 | 0.043 | 4.20E-08 | 359729 | 0.00008 | 30 | AUTS2 |
| rs1999292 | - | - | 21 | 22366346 | T | A | 0.600 | -0.100 | 0.018 | 4.48E-08 | 359729 | 0.00008 | 30 | PPIAP1, NCAM2 |
| rs2024471 | - | - | 16 | 53899008 | A | T | 0.496 | -0.106 | 0.018 | 3.06E-09 | 359729 | 0.0001 | 35 | FTO |
| rs2043962 | - | - | 18 | 53072319 | A | C | 0.871 | -0.168 | 0.027 | 4.66E-10 | 359729 | 0.00011 | 39 | TCF4 |
| rs2048719 | - | - | 12 | 43581323 | T | C | 0.444 | 0.106 | 0.018 | 4.50E-09 | 359729 | 0.0001 | 34 | RP11-118A3.1 |
| rs2246005 | - | - | 21 | 40060271 | A | G | 0.758 | 0.115 | 0.021 | 3.49E-08 | 359729 | 0.00008 | 30 | PCP4 |
| rs2307075 | - | - | 8 | 86388228 | A | C | 0.592 | -0.099 | 0.018 | 4.57E-08 | 359729 | 0.00008 | 30 | CA2 |
| rs2341569 | - | - | 12 | 124390681 | A | G | 0.344 | -0.102 | 0.019 | 4.77E-08 | 359729 | 0.00008 | 30 | DNAH10 |
| rs2427574 | - | - | 20 | 62584526 | C | A | 0.401 | 0.110 | 0.018 | 1.59E-09 | 359729 | 0.0001 | 36 | UCKL1 |
| rs2489364 | - | - | 9 | 103332321 | A | T | 0.617 | 0.106 | 0.018 | 7.35E-09 | 359729 | 0.00009 | 33 | TMEFF1 |
| rs249520 | - | - | 5 | 171184704 | A | G | 0.659 | 0.105 | 0.019 | 2.60E-08 | 359729 | 0.00009 | 31 | HMP19 |
| rs2798631 | - | - | 1 | 218611878 | A | G | 0.492 | 0.099 | 0.018 | 3.93E-08 | 359729 | 0.00008 | 30 | TGFB2 |
| rs2894602 | - | - | 2 | 227249802 | A | G | 0.763 | 0.122 | 0.021 | 8.57E-09 | 359729 | 0.00009 | 33 | NEU2 |
| rs2971154 | - | - | 12 | 23994841 | T | C | 0.408 | -0.119 | 0.018 | 4.96E-11 | 359729 | 0.00012 | 43 | SOX5 |
| rs3132442 | - | - | 6 | 31839494 | C | T | 0.507 | -0.138 | 0.018 | 8.17E-15 | 359729 | 0.00017 | 60 | SLC44A4 |
| rs34217742 | - | - | 19 | 37376830 | T | A | 0.125 | 0.151 | 0.027 | 2.56E-08 | 359729 | 0.00009 | 31 | ZNF345 |
| rs34465449 | - | - | 17 | 43511435 | C | A | 0.186 | -0.158 | 0.023 | 5.17E-12 | 359729 | 0.00013 | 48 | ARHGAP27 |
| rs34824085 | - | - | 8 | 110350921 | A | G | 0.171 | -0.135 | 0.024 | 1.27E-08 | 359729 | 0.00009 | 32 | ENY2 |
| rs35166564 | - | - | 19 | 2152018 | T | C | 0.390 | 0.103 | 0.018 | 1.92E-08 | 359729 | 0.00009 | 32 | AP3D1 |
| rs35184771 | - | - | 11 | 47475189 | G | T | 0.352 | -0.103 | 0.019 | 3.37E-08 | 359729 | 0.00008 | 30 | CELF1, RAPSN |
| rs35809681 | - | - | 16 | 2125490 | T | C | 0.105 | -0.166 | 0.029 | 1.52E-08 | 359729 | 0.00009 | 32 | TSC2 |
| rs36028059 | - | - | 16 | 84859672 | A | G | 0.427 | -0.101 | 0.018 | 2.18E-08 | 359729 | 0.00009 | 31 | CRISPLD2 |
| rs36112366 | - | - | 8 | 57138676 | T | G | 0.110 | -0.157 | 0.029 | 3.93E-08 | 359729 | 0.00008 | 30 | RP11-140I16.3 |
| rs3781295 | - | - | 10 | 104140602 | G | A | 0.368 | -0.110 | 0.018 | 2.30E-09 | 359729 | 0.0001 | 36 | GBF1 |
| rs3887753 | - | - | 1 | 227695517 | T | C | 0.178 | -0.152 | 0.024 | 3.25E-10 | 359729 | 0.00011 | 40 | TUBB8P9 |
| rs398745 | - | - | 14 | 36536181 | A | C | 0.411 | 0.102 | 0.018 | 2.15E-08 | 359729 | 0.00009 | 31 | RP11-116N8.4 |
| rs417614 | - | - | 2 | 40377084 | G | A | 0.260 | 0.111 | 0.020 | 4.50E-08 | 359729 | 0.00008 | 30 | SLC8A1 |
| rs4310973 | - | - | 18 | 20707622 | A | G | 0.786 | 0.172 | 0.022 | 3.54E-15 | 359729 | 0.00017 | 62 | CABLES1, RBBP8 |
| rs4379363 | - | - | 7 | 18953143 | T | C | 0.217 | 0.125 | 0.022 | 8.07E-09 | 359729 | 0.00009 | 33 | HDAC9 |
| rs4393380 | - | - | 12 | 48336311 | G | T | 0.155 | -0.144 | 0.025 | 5.53E-09 | 359729 | 0.00009 | 34 | - |
| rs4393510 | - | - | 14 | 105162257 | C | T | 0.574 | -0.100 | 0.018 | 3.36E-08 | 359729 | 0.00008 | 30 | INF2 |
| rs4517588 | - | - | 12 | 79538633 | A | G | 0.610 | -0.107 | 0.018 | 3.80E-09 | 359729 | 0.0001 | 35 | SYT1 |
| rs4696510 | - | - | 4 | 154772660 | T | A | 0.468 | -0.100 | 0.018 | 2.71E-08 | 359729 | 0.00009 | 31 | TOMM22P4 |
| rs4751671 | - | - | 10 | 116138744 | G | A | 0.531 | 0.101 | 0.018 | 1.82E-08 | 359729 | 0.00009 | 32 | AFAP1L2 |
| rs4783718 | - | - | 16 | 69547741 | T | C | 0.589 | 0.105 | 0.018 | 1.29E-08 | 359729 | 0.00009 | 32 | CYB5B |
| rs4795318 | - | - | 17 | 36949053 | C | T | 0.520 | 0.110 | 0.018 | 7.39E-10 | 359729 | 0.00011 | 38 | PIP4K2B |
| rs4802848 | - | - | 19 | 52218342 | G | C | 0.731 | 0.135 | 0.020 | 1.57E-11 | 359729 | 0.00013 | 45 | HAS1 |
| rs4869851 | - | - | 6 | 155613906 | A | G | 0.199 | 0.124 | 0.022 | 2.87E-08 | 359729 | 0.00009 | 31 | TFB1M |
| rs4945185 | - | - | 11 | 77318142 | G | A | 0.370 | -0.112 | 0.018 | 1.43E-09 | 359729 | 0.0001 | 37 | AQP11 |
| rs4994327 | - | - | 2 | 152351126 | T | C | 0.652 | -0.110 | 0.019 | 4.43E-09 | 359729 | 0.0001 | 34 | NEB |
| rs55706579 | - | - | 19 | 49382969 | G | A | 0.177 | -0.128 | 0.023 | 4.82E-08 | 359729 | 0.00008 | 30 | PPP1R15A |
| rs55915134 | - | - | 1 | 205641343 | C | G | 0.115 | 0.162 | 0.028 | 6.68E-09 | 359729 | 0.00009 | 34 | SLC45A3 |
| rs56074046 | - | - | 17 | 7358930 | G | A | 0.373 | -0.103 | 0.018 | 2.53E-08 | 359729 | 0.00009 | 31 | CHRNB1 |
| rs56100703 | - | - | 4 | 17780095 | C | A | 0.210 | -0.152 | 0.022 | 3.67E-12 | 359729 | 0.00013 | 48 | FAM184B |
| rs56223081 | - | - | 11 | 65961498 | G | T | 0.054 | -0.247 | 0.040 | 8.87E-10 | 359729 | 0.0001 | 38 | PACS1 |
| rs56403662 | - | - | 4 | 118988617 | T | A | 0.064 | -0.206 | 0.037 | 2.09E-08 | 359729 | 0.00009 | 31 | NDST3 |
| rs564340 | - | - | 6 | 20031359 | T | C | 0.229 | -0.117 | 0.021 | 3.34E-08 | 359729 | 0.00008 | 30 | RP1-130G2.1 |
| rs57884925 | rs7034200 | 0.9685 | 9 | 4285119 | C | G | 0.479 | 0.101 | 0.018 | 1.42E-08 | 359729 | 0.00009 | 32 | GLIS3 |
| rs58319813 | - | - | 17 | 27360504 | T | C | 0.153 | -0.140 | 0.025 | 1.60E-08 | 359729 | 0.00009 | 32 | PIPOX |
| rs6007580 | - | - | 22 | 45700514 | G | A | 0.259 | 0.112 | 0.020 | 3.27E-08 | 359729 | 0.00008 | 31 | FAM118A |
| rs6060355 | - | - | 20 | 33890061 | G | A | 0.366 | 0.234 | 0.019 | 1.84E-36 | 359729 | 0.00044 | 159 | UQCC1, FAM83C |
| rs6087571 | - | - | 20 | 32912091 | A | G | 0.137 | 0.147 | 0.026 | 2.00E-08 | 359729 | 0.00009 | 31 | RP11-101E14.3 |
| rs61780429 | - | - | 1 | 41457693 | T | A | 0.218 | 0.147 | 0.022 | 1.15E-11 | 359729 | 0.00013 | 46 | CTPS1 |
| rs62253602 | - | - | 3 | 52970877 | C | T | 0.341 | 0.108 | 0.019 | 7.98E-09 | 359729 | 0.00009 | 33 | SFMBT1 |
| rs62346126 | - | - | 4 | 145560166 | C | A | 0.814 | 0.134 | 0.023 | 4.71E-09 | 359729 | 0.0001 | 34 | HHIP-AS1, KRT18P51 |
| rs62621197 | - | - | 19 | 8670147 | C | T | 0.036 | -0.291 | 0.049 | 3.73E-09 | 359729 | 0.0001 | 35 | ADAMTS10 |
| rs6573308 | - | - | 14 | 60806976 | C | T | 0.387 | 0.102 | 0.018 | 2.58E-08 | 359729 | 0.00009 | 31 | CTD-2568P8.1 |
| rs6679313 | - | - | 1 | 170715110 | A | G | 0.364 | -0.103 | 0.019 | 3.15E-08 | 359729 | 0.00009 | 31 | PRRX1, HNRNPA1P46 |
| rs6731993 | - | - | 2 | 65642097 | A | T | 0.410 | -0.108 | 0.018 | 2.55E-09 | 359729 | 0.0001 | 36 | SPRED2 |
| rs6792762 | - | - | 3 | 38574491 | G | A | 0.418 | -0.103 | 0.018 | 1.32E-08 | 359729 | 0.00009 | 32 | EXOG |
| rs6864888 | - | - | 5 | 88273653 | G | T | 0.388 | -0.107 | 0.018 | 5.06E-09 | 359729 | 0.00009 | 34 | MEF2C-AS1 |
| rs6867409 | - | - | 5 | 103890096 | C | T | 0.465 | -0.099 | 0.018 | 2.85E-08 | 359729 | 0.00009 | 31 | RP11-6N13.1 |
| rs6882168 | - | - | 5 | 39402647 | C | T | 0.339 | -0.105 | 0.019 | 2.25E-08 | 359729 | 0.00009 | 31 | DAB2 |
| rs6902789 | - | - | 6 | 105358192 | G | A | 0.369 | 0.111 | 0.019 | 1.78E-09 | 359729 | 0.0001 | 36 | LIN28B-AS1 |
| rs7016366 | - | - | 8 | 74489718 | T | C | 0.201 | 0.125 | 0.022 | 1.60E-08 | 359729 | 0.00009 | 32 | STAU2 |
| rs701810 | - | - | 10 | 98974849 | C | T | 0.325 | 0.105 | 0.019 | 3.71E-08 | 359729 | 0.00008 | 30 | ARHGAP19-SLIT1 |
| rs7084705 | - | - | 10 | 79596675 | A | G | 0.341 | -0.109 | 0.019 | 6.66E-09 | 359729 | 0.00009 | 34 | ;DLG5 |
| rs72751292 | - | - | 15 | 96898502 | A | G | 0.394 | 0.103 | 0.019 | 4.38E-08 | 359729 | 0.00008 | 30 | RP11-522B15.3 |
| rs7314075 | - | - | 12 | 14945417 | G | A | 0.152 | -0.141 | 0.025 | 2.66E-08 | 359729 | 0.00009 | 31 | WBP11 |
| rs75069534 | - | - | 2 | 199196373 | G | A | 0.106 | 0.182 | 0.029 | 7.04E-10 | 359729 | 0.00011 | 38 | LINC01923 |
| rs7608976 | - | - | 2 | 25075281 | G | A | 0.428 | -0.128 | 0.018 | 1.14E-12 | 359729 | 0.00014 | 51 | ADCY3 |
| rs76310549 | - | - | 11 | 74272884 | C | T | 0.095 | -0.182 | 0.031 | 3.27E-09 | 359729 | 0.0001 | 35 | POLD3 |
| rs7633561 | - | - | 3 | 196785820 | T | C | 0.451 | 0.101 | 0.018 | 1.82E-08 | 359729 | 0.00009 | 32 | DLG1 |
| rs7636670 | - | - | 3 | 193486579 | A | C | 0.571 | 0.101 | 0.018 | 3.56E-08 | 359729 | 0.00008 | 30 | RP11-528A4.3 |
| rs76895963 | - | - | 12 | 4384844 | T | G | 0.021 | 0.445 | 0.068 | 7.71E-11 | 359729 | 0.00012 | 42 | CCND2, CCND2-AS1 |
| rs7701967 | - | - | 5 | 130059750 | G | A | 0.346 | 0.103 | 0.019 | 4.25E-08 | 359729 | 0.00008 | 30 | CTB-1I21.1 |
| rs7741360 | - | - | 6 | 7684917 | G | A | 0.465 | 0.113 | 0.018 | 5.00E-10 | 359729 | 0.00011 | 39 | TMEM14C |
| rs7766407 | - | - | 6 | 66885658 | C | A | 0.590 | 0.099 | 0.018 | 4.41E-08 | 359729 | 0.00008 | 30 | RNU7-66P |
| rs7790322 | - | - | 7 | 2830498 | C | T | 0.417 | -0.102 | 0.018 | 1.65E-08 | 359729 | 0.00009 | 32 | GNA12 |
| rs7795051 | - | - | 7 | 150504341 | A | T | 0.268 | 0.121 | 0.020 | 2.06E-09 | 359729 | 0.0001 | 36 | TMEM176A, AOC1 |
| rs7909837 | - | - | 10 | 81228041 | T | C | 0.405 | 0.103 | 0.019 | 3.30E-08 | 359729 | 0.00008 | 31 | ZCCHC24 |
| rs8012800 | - | - | 14 | 80597551 | G | C | 0.256 | 0.118 | 0.020 | 8.53E-09 | 359729 | 0.00009 | 33 | DIO2 |
| rs8049014 | - | - | 16 | 24698903 | G | A | 0.753 | 0.125 | 0.021 | 1.66E-09 | 359729 | 0.0001 | 36 | TNRC6A |
| rs806292 | - | - | 13 | 50811031 | A | G | 0.533 | -0.104 | 0.018 | 5.18E-09 | 359729 | 0.00009 | 34 | DLEU1 |
| rs8069671 | - | - | 17 | 36826936 | G | T | 0.390 | -0.102 | 0.018 | 2.58E-08 | 359729 | 0.00009 | 31 | EPOP |
| rs8180765 | - | - | 7 | 4680614 | A | G | 0.218 | -0.119 | 0.022 | 4.00E-08 | 359729 | 0.00008 | 30 | FOXK1 |
| rs911641 | - | - | 20 | 13259045 | C | G | 0.369 | 0.102 | 0.019 | 4.32E-08 | 359729 | 0.00008 | 30 | ISM1 |
| rs9284832 | - | - | 3 | 13810540 | G | C | 0.731 | -0.112 | 0.020 | 2.67E-08 | 359729 | 0.00009 | 31 | LINC00690 |
| rs9400284 | - | - | 6 | 109810581 | G | C | 0.254 | 0.116 | 0.020 | 1.51E-08 | 359729 | 0.00009 | 32 | AK9 |
| rs945890 | - | - | 6 | 130321899 | A | T | 0.714 | -0.149 | 0.020 | 4.17E-14 | 359729 | 0.00016 | 57 | RP11-394G3.2 |
| rs9469813 | - | - | 6 | 34569814 | T | C | 0.121 | -0.151 | 0.027 | 3.45E-08 | 359729 | 0.00008 | 30 | C6orf106 |
| rs9894577 | - | - | 17 | 43223292 | G | A | 0.317 | -0.127 | 0.019 | 3.24E-11 | 359729 | 0.00012 | 44 | ACBD4, HEXIM1 |
| rs9931120 | - | - | 16 | 89501357 | A | G | 0.476 | -0.100 | 0.018 | 2.43E-08 | 359729 | 0.00009 | 31 | ANKRD11 |
| rs9952698 | - | - | 18 | 13021630 | G | A | 0.568 | -0.100 | 0.018 | 2.62E-08 | 359729 | 0.00009 | 31 | CEP192 |
| rs9987012 | - | - | 7 | 39090684 | T | G | 0.764 | -0.118 | 0.021 | 1.84E-08 | 359729 | 0.00009 | 32 | POU6F2 |
| rs9991590 | - | - | 4 | 30647963 | T | C | 0.721 | 0.110 | 0.020 | 3.46E-08 | 359729 | 0.00008 | 30 | snoU13 |
| rs7034200* | - | - | 9 | 4289050 | G | A | 0.478 | 0.099 | 0.018 | 2.6E-08 | 359729 | 0.00009 | 31 | RP11-474P2.6 |

Abbreviations:SNPs, Single-nucleotide polymorphiss. CHR, chromosome. EA, effect allele.EAF, effect allele frequency. SE, standard error. R², explained variation by SNPs.

*rs7034200 was used as a proxy for rs57884925 when evaluate the association between right handgrip strength and heart failure or atrial fibrillation.

# Supplementary Table 3.Genome-wide significant SNPs for left handgrip strength.

| SNP | Proxy SNP | r2 for proxy | CHR | Position | OA | EA | EAF | BETA | SE | P-value | N | R^2^ | F statistic | genes |
| --- | --- | --- | --- | --- | --- | --- | --- | --- | --- | --- | --- | --- | --- | --- |
| rs10043473 | - | - | 5 | 67799515 | A | G | 0.400 | -0.101 | 0.018 | 2.62E-08 | 359704 | 0.00009 | 31 | JMY |
| rs10176878 | - | - | 2 | 59952274 | T | C | 0.192 | -0.137 | 0.023 | 1.67E-09 | 359704 | 0.0001 | 36 | AC007131.2 |
| rs10403906 | - | - | 19 | 37376756 | G | A | 0.478 | -0.108 | 0.018 | 1.30E-09 | 359704 | 0.0001 | 37 | ZNF345 |
| rs10445885 | - | - | 2 | 44116100 | C | T | 0.472 | 0.116 | 0.018 | 1.41E-10 | 359704 | 0.00011 | 41 | LRPPRC |
| rs10493174 | - | - | 1 | 54038536 | C | A | 0.216 | -0.128 | 0.022 | 4.85E-09 | 359704 | 0.0001 | 34 | GLIS1 |
| rs10496731 | - | - | 2 | 135597628 | T | G | 0.363 | 0.128 | 0.019 | 6.45E-12 | 359704 | 0.00013 | 47 | ACMSD, CCNT2-AS1 |
| rs10753823 | - | - | 1 | 170798444 | A | G | 0.329 | -0.106 | 0.019 | 2.39E-08 | 359704 | 0.00009 | 31 | PRRX1 |
| rs10798482 | - | - | 1 | 176772439 | C | T | 0.459 | 0.124 | 0.018 | 4.53E-12 | 359704 | 0.00013 | 48 | PAPPA2 |
| rs10851633 | - | - | 15 | 58323065 | C | T | 0.375 | -0.105 | 0.018 | 1.01E-08 | 359704 | 0.00009 | 33 | ALDH1A2 |
| rs10883733 | - | - | 10 | 104302203 | T | A | 0.526 | 0.121 | 0.019 | 1.14E-10 | 359704 | 0.00012 | 42 | SUFU |
| rs10916148 | - | - | 1 | 227629576 | T | A | 0.210 | -0.134 | 0.022 | 1.48E-09 | 359704 | 0.0001 | 37 | BTF3P9 |
| rs10988217 | - | - | 9 | 131888116 | A | G | 0.604 | -0.101 | 0.018 | 3.40E-08 | 359704 | 0.00008 | 30 | PTPA |
| rs11003014 | - | - | 10 | 81231387 | A | G | 0.159 | 0.141 | 0.024 | 7.98E-09 | 359704 | 0.00009 | 33 | ZCCHC24 |
| rs11067228 | - | - | 12 | 115094260 | A | G | 0.449 | -0.100 | 0.018 | 2.14E-08 | 359704 | 0.00009 | 31 | OSTF1P1, Y_RNA |
| rs11073613 | - | - | 15 | 85185893 | G | A | 0.485 | 0.099 | 0.018 | 3.77E-08 | 359704 | 0.00008 | 30 | WDR73 |
| rs11121529 | - | - | 1 | 10271688 | C | G | 0.114 | -0.154 | 0.028 | 3.63E-08 | 359704 | 0.00008 | 30 | KIF1B |
| rs112570672 | - | - | 4 | 17914257 | G | A | 0.044 | -0.270 | 0.043 | 5.45E-10 | 359704 | 0.00011 | 39 | LCORL, KRT18P63 |
| rs113602522 | - | - | 15 | 56798775 | C | A | 0.227 | -0.120 | 0.022 | 2.70E-08 | 359704 | 0.00009 | 31 | RP11-1129I3.1 |
| rs115771255 | - | - | 3 | 132119691 | A | T | 0.100 | 0.165 | 0.030 | 3.01E-08 | 359704 | 0.00009 | 31 | NIP7P2 |
| rs11676702 | - | - | 2 | 68410587 | T | C | 0.080 | -0.187 | 0.033 | 1.27E-08 | 359704 | 0.00009 | 32 | PPP3R1 |
| rs116825011 | - | - | 6 | 29156334 | G | A | 0.016 | 0.387 | 0.071 | 4.51E-08 | 359704 | 0.00008 | 30 | ZFP57 |
| rs1177590 | - | - | 14 | 36632555 | C | T | 0.704 | -0.106 | 0.019 | 4.93E-08 | 359704 | 0.00008 | 30 | LINC00609 |
| rs117844066 | - | - | 16 | 72001521 | G | C | 0.146 | 0.139 | 0.025 | 4.17E-08 | 359704 | 0.00008 | 30 | PKD1L3 |
| rs11826567 | - | - | 11 | 74279293 | G | A | 0.255 | 0.137 | 0.020 | 2.51E-11 | 359704 | 0.00012 | 45 | POLD3 |
| rs1205346 | - | - | 20 | 32914269 | C | T | 0.511 | -0.118 | 0.018 | 3.19E-11 | 359704 | 0.00012 | 44 | RP11-101E14.3 |
| rs12146743 | - | - | 12 | 14965356 | T | C | 0.371 | -0.136 | 0.018 | 1.54E-13 | 359704 | 0.00015 | 55 | C12orf60 |
| rs12153391 | - | - | 5 | 171203438 | C | A | 0.246 | -0.118 | 0.021 | 1.15E-08 | 359704 | 0.00009 | 33 | CTB-78H18.1 |
| rs1217456 | - | - | 2 | 201092279 | C | T | 0.422 | 0.100 | 0.018 | 3.49E-08 | 359704 | 0.00008 | 30 | SPATS2L |
| rs12356830 | - | - | 10 | 130830938 | T | C | 0.354 | 0.119 | 0.019 | 3.52E-10 | 359704 | 0.00011 | 39 | RP11-442O18.1 |
| rs12435835 | - | - | 14 | 65499909 | T | G | 0.516 | -0.099 | 0.018 | 2.98E-08 | 359704 | 0.00009 | 31 | CHURC1-FNTB |
| rs12889267 | - | - | 14 | 21542766 | A | G | 0.168 | -0.149 | 0.024 | 3.62E-10 | 359704 | 0.00011 | 39 | ARHGEF40 |
| rs12902985 | - | - | 15 | 99166519 | G | T | 0.333 | 0.105 | 0.019 | 4.21E-08 | 359704 | 0.00008 | 30 | RP11-35O15.1 |
| rs12905519 | - | - | 15 | 77399549 | T | C | 0.110 | -0.160 | 0.028 | 2.03E-08 | 359704 | 0.00009 | 31 | PEAK1 |
| rs13023088 | - | - | 2 | 100576304 | C | T | 0.148 | 0.154 | 0.025 | 8.91E-10 | 359704 | 0.0001 | 38 | AFF3 |
| rs13091492 | - | - | 3 | 81891476 | A | G | 0.375 | -0.102 | 0.018 | 2.59E-08 | 359704 | 0.00009 | 31 | RP11-359D24.1 |
| rs13150083 | - | - | 4 | 30863804 | G | A | 0.251 | -0.123 | 0.021 | 2.46E-09 | 359704 | 0.0001 | 36 | PCDH7 |
| rs1317490 | - | - | 1 | 227172518 | C | T | 0.282 | -0.116 | 0.020 | 8.45E-09 | 359704 | 0.00009 | 33 | COQ8A |
| rs13213814 | - | - | 6 | 15519376 | T | C | 0.259 | -0.113 | 0.020 | 3.24E-08 | 359704 | 0.00008 | 31 | JARID2 |
| rs13265437 | - | - | 8 | 4847438 | G | C | 0.382 | -0.107 | 0.018 | 6.32E-09 | 359704 | 0.00009 | 34 | CSMD1 |
| rs13298297 | - | - | 9 | 119264108 | G | A | 0.230 | 0.122 | 0.021 | 1.04E-08 | 359704 | 0.00009 | 33 | ASTN2 |
| rs13331451 | - | - | 16 | 2125788 | C | T | 0.071 | -0.198 | 0.035 | 1.47E-08 | 359704 | 0.00009 | 32 | TSC2 |
| rs141615456 | - | - | 3 | 135472797 | C | A | 0.085 | -0.180 | 0.032 | 2.02E-08 | 359704 | 0.00009 | 31 | KRT18P35 |
| rs1418653 | - | - | 1 | 205646278 | C | A | 0.491 | -0.098 | 0.018 | 4.24E-08 | 359704 | 0.00008 | 30 | SLC45A3 |
| rs142925250 | - | - | 17 | 43563606 | A | T | 0.201 | -0.158 | 0.023 | 4.90E-12 | 359704 | 0.00013 | 48 | PLEKHM1 |
| rs1480474 | - | - | 12 | 66326943 | A | G | 0.414 | -0.116 | 0.018 | 1.39E-10 | 359704 | 0.00011 | 41 | HMGA2 |
| rs16910750 | - | - | 9 | 99084471 | G | C | 0.159 | 0.137 | 0.024 | 2.23E-08 | 359704 | 0.00009 | 31 | SLC35D2 |
| rs17140345 | - | - | 7 | 18977511 | T | G | 0.166 | 0.132 | 0.024 | 2.89E-08 | 359704 | 0.00009 | 31 | HDAC9 |
| rs17282763 | - | - | 7 | 82520166 | T | C | 0.296 | 0.110 | 0.020 | 1.78E-08 | 359704 | 0.00009 | 32 | PCLO |
| rs17536644 | - | - | 7 | 120721025 | A | G | 0.440 | 0.098 | 0.018 | 4.49E-08 | 359704 | 0.00008 | 30 | CPED1 |
| rs1853927 | - | - | 13 | 50875574 | G | A | 0.498 | -0.101 | 0.018 | 1.77E-08 | 359704 | 0.00009 | 32 | DLEU1 |
| rs185490114 | - | - | 6 | 31035118 | G | A | 0.222 | -0.137 | 0.021 | 1.61E-10 | 359704 | 0.00011 | 41 | TBC1D22B |
| rs1862901 | - | - | 2 | 23886149 | T | C | 0.690 | 0.111 | 0.019 | 1.07E-08 | 359704 | 0.00009 | 33 | KLHL29 |
| rs187673257 | - | - | 1 | 160264868 | C | T | 0.032 | -0.298 | 0.051 | 4.39E-09 | 359704 | 0.0001 | 34 | COPA |
| rs1884447 | - | - | 1 | 185021410 | G | A | 0.399 | 0.109 | 0.018 | 1.70E-09 | 359704 | 0.0001 | 36 | RNF2 |
| rs1918202 | - | - | 12 | 79584218 | A | G | 0.431 | 0.113 | 0.018 | 3.02E-10 | 359704 | 0.00011 | 40 | SYT1 |
| rs1971940 | - | - | 5 | 58460599 | C | T | 0.687 | -0.106 | 0.019 | 3.41E-08 | 359704 | 0.00008 | 30 | PDE4D |
| rs2035609 | - | - | 8 | 78808755 | A | G | 0.305 | -0.107 | 0.019 | 3.78E-08 | 359704 | 0.00008 | 30 | RP11-91P17.1 |
| rs2045556 | - | - | 12 | 46673433 | A | G | 0.677 | 0.120 | 0.019 | 3.30E-10 | 359704 | 0.00011 | 39 | SLC38A1, SLC38A2 |
| rs2163971 | - | - | 3 | 85428726 | T | C | 0.552 | -0.105 | 0.018 | 5.84E-09 | 359704 | 0.00009 | 34 | CADM2 |
| rs218341 | - | - | 1 | 23294153 | G | C | 0.327 | -0.114 | 0.019 | 1.93E-09 | 359704 | 0.0001 | 36 | LACTBL1 |
| rs224390 | - | - | 20 | 34105975 | G | A | 0.223 | 0.147 | 0.021 | 7.04E-12 | 359704 | 0.00013 | 47 | CEP250 |
| rs2291119 | - | - | 11 | 47298201 | C | T | 0.291 | -0.109 | 0.020 | 2.52E-08 | 359704 | 0.00009 | 31 | MADD |
| rs2355371 | - | - | 16 | 19994743 | C | G | 0.311 | 0.111 | 0.019 | 9.62E-09 | 359704 | 0.00009 | 33 | GPR139 |
| rs2431108 | - | - | 5 | 103947968 | T | C | 0.329 | -0.104 | 0.019 | 3.97E-08 | 359704 | 0.00008 | 30 | RP11-6N13.1 |
| rs2565689 | - | - | 2 | 218110548 | T | A | 0.747 | 0.114 | 0.021 | 3.05E-08 | 359704 | 0.00009 | 31 | SERPINE2 |
| rs2807504 | - | - | 6 | 73058954 | G | A | 0.721 | 0.109 | 0.020 | 3.72E-08 | 359704 | 0.00008 | 30 | RIMS1 |
| rs28409768 | - | - | 15 | 99193524 | C | T | 0.031 | -0.288 | 0.052 | 2.65E-08 | 359704 | 0.00009 | 31 | IGF1R |
| rs2974438 | - | - | 5 | 168250903 | G | A | 0.210 | -0.120 | 0.022 | 4.09E-08 | 359704 | 0.00008 | 30 | SLIT3 |
| rs34159998 | - | - | 2 | 179292821 | C | T | 0.046 | -0.236 | 0.043 | 4.13E-08 | 359704 | 0.00008 | 30 | PRKRA |
| rs343957 | - | - | 2 | 44942384 | C | G | 0.594 | 0.107 | 0.018 | 3.80E-09 | 359704 | 0.0001 | 35 | CAMKMT |
| rs34824085 | - | - | 8 | 110350921 | A | G | 0.171 | -0.132 | 0.024 | 2.40E-08 | 359704 | 0.00009 | 31 | ENY2 |
| rs35236379 | - | - | 10 | 5727292 | G | T | 0.143 | 0.139 | 0.025 | 4.96E-08 | 359704 | 0.00008 | 30 | FAM208B |
| rs36086419 | - | - | 11 | 133761715 | G | C | 0.268 | 0.115 | 0.020 | 1.18E-08 | 359704 | 0.00009 | 33 | MIR4697HG |
| rs3781295 | - | - | 10 | 104140602 | G | A | 0.368 | -0.106 | 0.018 | 8.88E-09 | 359704 | 0.00009 | 33 | GBF1 |
| rs3829814 | - | - | 7 | 140426257 | A | G | 0.563 | -0.100 | 0.018 | 4.07E-08 | 359704 | 0.00008 | 30 | BRAF |
| rs3850625 | - | - | 1 | 201016296 | G | A | 0.119 | -0.163 | 0.027 | 3.20E-09 | 359704 | 0.0001 | 35 | CACNA1S |
| rs4128846 | - | - | 7 | 46541914 | T | C | 0.623 | -0.101 | 0.018 | 3.76E-08 | 359704 | 0.00008 | 30 | AC004869.2 |
| rs417591 | - | - | 2 | 40400312 | A | G | 0.215 | 0.130 | 0.022 | 2.12E-09 | 359704 | 0.0001 | 36 | SLC8A1 |
| rs4310973 | - | - | 18 | 20707622 | A | G | 0.786 | 0.175 | 0.022 | 1.21E-15 | 359704 | 0.00018 | 64 | CABLES1, RBBP8 |
| rs4393380 | - | - | 12 | 48336311 | G | T | 0.155 | -0.141 | 0.025 | 1.21E-08 | 359704 | 0.00009 | 32 | - |
| rs4705885 | - | - | 5 | 130423559 | G | A | 0.376 | 0.109 | 0.019 | 4.18E-09 | 359704 | 0.0001 | 35 | CTB-1I21.1 |
| rs4783718 | - | - | 16 | 69547741 | T | C | 0.589 | 0.123 | 0.018 | 2.79E-11 | 359704 | 0.00012 | 44 | CYB5B |
| rs4784328 | - | - | 16 | 53910195 | T | C | 0.467 | -0.099 | 0.018 | 2.70E-08 | 359704 | 0.00009 | 31 | FTO |
| rs4795318 | - | - | 17 | 36949053 | C | T | 0.520 | 0.118 | 0.018 | 4.22E-11 | 359704 | 0.00012 | 44 | PIP4K2B |
| rs4797782 | - | - | 18 | 13483798 | T | C | 0.422 | -0.100 | 0.018 | 2.92E-08 | 359704 | 0.00009 | 31 | LDLRAD4 |
| rs4802848 | - | - | 19 | 52218342 | G | C | 0.731 | 0.130 | 0.020 | 9.23E-11 | 359704 | 0.00012 | 42 | HAS1 |
| rs4903247 | - | - | 14 | 75073657 | A | G | 0.459 | 0.107 | 0.018 | 1.93E-09 | 359704 | 0.0001 | 36 | - |
| rs491347 | - | - | 11 | 68169688 | G | A | 0.742 | 0.117 | 0.020 | 9.71E-09 | 359704 | 0.00009 | 33 | LRP5 |
| rs4994327 | - | - | 2 | 152351126 | T | C | 0.652 | -0.105 | 0.019 | 2.22E-08 | 359704 | 0.00009 | 31 | NEB |
| rs553108 | - | - | 6 | 31840455 | A | G | 0.577 | -0.130 | 0.018 | 4.53E-13 | 359704 | 0.00015 | 52 | SLC44A4 |
| rs56100703 | - | - | 4 | 17780095 | C | A | 0.210 | -0.141 | 0.022 | 1.10E-10 | 359704 | 0.00012 | 42 | FAM184B |
| rs56402185 | - | - | 10 | 79585848 | G | A | 0.339 | -0.106 | 0.019 | 2.12E-08 | 359704 | 0.00009 | 31 | DLG5 |
| rs58319813 | - | - | 17 | 27360504 | T | C | 0.153 | -0.135 | 0.025 | 4.49E-08 | 359704 | 0.00008 | 30 | PIPOX |
| rs599550 | - | - | 18 | 53252388 | G | A | 0.848 | -0.165 | 0.025 | 2.87E-11 | 359704 | 0.00012 | 44 | TCF4 |
| rs60750824 | - | - | 7 | 99927436 | A | G | 0.185 | -0.129 | 0.023 | 4.17E-08 | 359704 | 0.00008 | 30 | PMS2P1 |
| rs61818099 | - | - | 1 | 190961337 | G | A | 0.116 | 0.153 | 0.028 | 3.27E-08 | 359704 | 0.00008 | 31 | HNRNPA1P46 |
| rs62253602 | - | - | 3 | 52970877 | C | T | 0.341 | 0.118 | 0.019 | 4.10E-10 | 359704 | 0.00011 | 39 | SFMBT1 |
| rs62332075 | - | - | 4 | 154834701 | A | G | 0.487 | -0.101 | 0.018 | 1.79E-08 | 359704 | 0.00009 | 32 | TOMM22P4 |
| rs62346126 | - | - | 4 | 145560166 | C | A | 0.814 | 0.128 | 0.023 | 2.44E-08 | 359704 | 0.00009 | 31 | HHIP-AS1, KRT18P51 |
| rs6433478 | - | - | 2 | 175241482 | T | C | 0.546 | 0.099 | 0.018 | 3.35E-08 | 359704 | 0.00008 | 30 | CIR1 |
| rs6503398 | - | - | 17 | 42775217 | T | G | 0.523 | 0.124 | 0.018 | 3.94E-12 | 359704 | 0.00013 | 48 | RP11-1072C15.6 |
| rs6592728 | - | - | 11 | 77247260 | A | G | 0.372 | -0.113 | 0.018 | 7.85E-10 | 359704 | 0.00011 | 38 | - |
| rs6731993 | - | - | 2 | 65642097 | A | T | 0.410 | -0.111 | 0.018 | 8.92E-10 | 359704 | 0.0001 | 38 | SPRED2 |
| rs6882168 | - | - | 5 | 39402647 | C | T | 0.339 | -0.109 | 0.019 | 7.24E-09 | 359704 | 0.00009 | 33 | DAB2 |
| rs701810 | - | - | 10 | 98974849 | C | T | 0.325 | 0.106 | 0.019 | 2.55E-08 | 359704 | 0.00009 | 31 | ARHGAP19-SLIT1 |
| rs71351952 | - | - | 20 | 47523789 | C | T | 0.249 | 0.121 | 0.021 | 4.87E-09 | 359704 | 0.0001 | 34 | CSE1L |
| rs71528371 | - | - | 8 | 135684638 | C | T | 0.147 | 0.137 | 0.025 | 4.60E-08 | 359704 | 0.00008 | 30 | ZFAT |
| rs7166220 | - | - | 15 | 74094248 | G | T | 0.219 | 0.128 | 0.022 | 3.07E-09 | 359704 | 0.0001 | 35 | C15orf59-AS1 |
| rs7174985 | - | - | 15 | 74274948 | T | G | 0.332 | -0.107 | 0.019 | 1.92E-08 | 359704 | 0.00009 | 32 | STOML1 |
| rs7249081 | - | - | 19 | 2157167 | T | C | 0.477 | 0.100 | 0.018 | 2.48E-08 | 359704 | 0.00009 | 31 | AP3D1 |
| rs72686716 | - | - | 4 | 102735510 | T | C | 0.105 | -0.212 | 0.030 | 1.81E-12 | 359704 | 0.00014 | 50 | BANK1 |
| rs72845891 | - | - | 17 | 61696759 | G | A | 0.347 | -0.107 | 0.019 | 1.67E-08 | 359704 | 0.00009 | 32 | MAP3K3, TACO1 |
| rs7301953 | - | - | 12 | 124405871 | G | A | 0.311 | -0.120 | 0.019 | 3.84E-10 | 359704 | 0.00011 | 39 | DNAH10, CCDC92 |
| rs75069534 | - | - | 2 | 199196373 | G | A | 0.106 | 0.169 | 0.029 | 8.88E-09 | 359704 | 0.00009 | 33 | LINC01923 |
| rs75659664 | - | - | 10 | 63557893 | G | A | 0.037 | -0.259 | 0.047 | 3.40E-08 | 359704 | 0.00008 | 30 | RP11-491H19.1 |
| rs7576689 | - | - | 2 | 42218358 | C | T | 0.219 | 0.128 | 0.022 | 3.24E-09 | 359704 | 0.0001 | 35 | AC013480.2 |
| rs7608976 | - | - | 2 | 25075281 | G | A | 0.428 | -0.141 | 0.018 | 5.91E-15 | 359704 | 0.00017 | 61 | ADCY3 |
| rs76895963 | - | - | 12 | 4384844 | T | G | 0.021 | 0.420 | 0.068 | 8.16E-10 | 359704 | 0.0001 | 38 | CCND2, CCND2-AS1 |
| rs7756651 | - | - | 6 | 7692576 | C | G | 0.498 | 0.113 | 0.018 | 3.43E-10 | 359704 | 0.00011 | 39 | TMEM14C |
| rs7761910 | - | - | 6 | 155553832 | C | T | 0.307 | -0.108 | 0.019 | 2.62E-08 | 359704 | 0.00009 | 31 | TIAM2 |
| rs7795051 | - | - | 7 | 150504341 | A | T | 0.268 | 0.135 | 0.020 | 2.66E-11 | 359704 | 0.00012 | 44 | TMEM176A, AOC1 |
| rs78743523 | - | - | 8 | 49680347 | G | C | 0.030 | -0.288 | 0.053 | 4.25E-08 | 359704 | 0.00008 | 30 | EFCAB1 |
| rs79177013 | - | - | 11 | 65733465 | G | A | 0.060 | -0.205 | 0.038 | 4.88E-08 | 359704 | 0.00008 | 30 | SART1 |
| rs8023263 | - | - | 15 | 51517597 | G | T | 0.528 | 0.098 | 0.018 | 4.08E-08 | 359704 | 0.00008 | 30 | MIR4713HG, CYP19A1 |
| rs8056535 | - | - | 16 | 29960468 | G | A | 0.458 | 0.099 | 0.018 | 2.98E-08 | 359704 | 0.00009 | 31 | TMEM219 |
| rs8069671 | - | - | 17 | 36826936 | G | T | 0.390 | -0.108 | 0.018 | 2.77E-09 | 359704 | 0.0001 | 35 | EPOP |
| rs8101782 | - | - | 19 | 12507992 | A | C | 0.705 | 0.115 | 0.021 | 2.68E-08 | 359704 | 0.00009 | 31 | ZNF799 |
| rs8138982 | - | - | 22 | 40610398 | C | A | 0.277 | 0.109 | 0.020 | 4.00E-08 | 359704 | 0.00008 | 30 | TNRC6B |
| rs8180765 | - | - | 7 | 4680614 | A | G | 0.218 | -0.128 | 0.022 | 4.32E-09 | 359704 | 0.0001 | 34 | FOXK1 |
| rs833520 | - | - | 18 | 46564559 | T | C | 0.792 | 0.121 | 0.022 | 4.15E-08 | 359704 | 0.00008 | 30 | DYM |
| rs889292 | - | - | 5 | 52850891 | T | C | 0.766 | 0.115 | 0.021 | 4.19E-08 | 359704 | 0.00008 | 30 | AC116606.1 |
| rs9284832 | - | - | 3 | 13810540 | G | C | 0.731 | -0.113 | 0.020 | 2.08E-08 | 359704 | 0.00009 | 31 | LINC00690 |
| rs9396861 | - | - | 6 | 18404133 | C | A | 0.600 | -0.119 | 0.019 | 1.47E-10 | 359704 | 0.00011 | 41 | RNF144B |
| rs9436117 | - | - | 1 | 150454885 | A | G | 0.753 | -0.113 | 0.021 | 4.58E-08 | 359704 | 0.00008 | 30 | TARS2 |
| rs945890 | - | - | 6 | 130321899 | A | T | 0.713 | -0.136 | 0.020 | 4.79E-12 | 359704 | 0.00013 | 48 | RP11-394G3.2 |
| rs9987012 | - | - | 7 | 39090684 | T | G | 0.764 | -0.121 | 0.021 | 1.01E-08 | 359704 | 0.00009 | 33 | POU6F2 |

Abbreviations:SNPs, Single-nucleotide polymorphiss. CHR, chromosome. EA, effect allele. OA, other allele. EAF, effect allele frequency. SE, standard error. R², explained variation by SNPs.

# Supplemental Table 4. Evidence of association (p<5*10-6) of the SNPs used as genetic variants for Mendelian randomization analyses of right hangrip strength with confounders or CVDs in the PhenoScanner and the GWAS catalog.

| SNP | GWAS catalog traits linked to this gene | PhenoScanner traits linked to this gene | Excluded from complementary analysis |
| --- | --- | --- | --- |
| rs10041126 | NA | Impedance of arm(UKBB);Whole body fat-free mass(UKBB) | NO |
| rs10176878 | NA | NA | NO |
| rs10185503 | NA | Weight(UKBB);Height(UKBB);fat-free mass(UKBB); body water mass(UKBB) | NO |
| rs10273327 | NA | Height(UKBB);fat-free mass(UKBB); body water mass(UKBB) | NO |
| rs10278546 | NA | Diastolic blood pressure(UKBB);Height(UKBB);Heart rate(UKBB) | YES(hypertension) |
| rs10445885 | NA | NA | NO |
| rs1047437 | NA | fat-free mass(UKBB); body water mass(UKBB) | NO |
| rs10496731 | NA | Diastolic blood pressure(UKBB);Systolic blood pressure(UKBB);body fat mass(UKBB) | YES(hypertension) |
| rs1065778 | NA | Height(UKBB);Hip circumference(UKBB) | NO |
| rs1076635 | NA | fat-free mass(UKBB) | NO |
| rs10788958 | NA | NA | NO |
| rs10797937 | NA | Height(UKBB);fat-free mass(UKBB);water mass(UKBB);Weight(UKBB);Hip circumference(UKBB) | NO |
| rs10851633 | NA | NA | NO |
| rs10998287 | NA | Height(UKBB); Fat-free mass(UKBB);Water mass(UKBB);Sleep duration(UKBB) | NO |
| rs11067228 | NA | Prostate specific antigen levels(PMID:28139693);Height(PMID:28146470) | NO |
| rs11072542 | NA | Height(PMID:25282103) | NO |
| rs11121529 | mean platelet volume | Height(UKBB);Asthma(UKBB) | NO |
| rs11125160 | NA | Height(UKBB); Fat-free mass(UKBB);Alcohol intake frequency(UKBB);Birth weight(UKBB) | YES(drinking) |
| rs11135316 | NA | NA | NO |
| rs112570672 | NA | Height(UKBB); Fat-free mass(UKBB);Water mass(UKBB);Forced vital capacity(UKBB);Weight(UKBB) | NO |
| rs112852122 | body mass index | Height(UKBB); Forced vital capacity(UKBB) | YES(BMI) |
| rs1138120 | NA | NA | NO |
| rs113835839 | NA | NA | NO |
| rs11546878 | body mass index;HDL | Height(UKBB); Fat-free mass(UKBB);Water mass(UKBB);Weight(UKBB);Body mass index(PMID:29273807);Hip circumference(UKBB) | YES(BMI) |
| rs115771255 | NA | Coronary artery disease(PMID:29212778) | YES(CAD) |
| rs11584359 | NA | Height(UKBB); Fat-free mass(UKBB);Water mass(UKBB);Weight(UKBB);Hip circumference(UKBB) | NO |
| rs116222218 | NA | Height(UKBB); Fat-free mass(UKBB);Water mass(UKBB);Forced vital capacity(UKBB);Weight(UKBB) | NO |
| rs11659241 | NA | Height(UKBB); Fat-free mass(UKBB);Water mass(UKBB);Weight(UKBB);Hip circumference(UKBB) | NO |
| rs116725229 | NA | NA | NO |
| rs11676702 | NA | Height(UKBB); Fat-free mass(UKBB);Water mass(UKBB) | NO |
| rs116782923 | NA | Height(UKBB) | NO |
| rs116922558 | NA | Height(UKBB);Coronary artery disease(PMID:26343387) | YES(CAD) |
| rs11810706 | NA | Weight(UKBB);Hip circumference(UKBB);Body mass index in females(PMID:23754948) | YES(BMI) |
| rs11854314 | NA | Height(UKBB);Forced vital capacity(UKBB) | NO |
| rs11857557 | NA | NA | NO |
| rs11887431 | NA | Sitting height(UKBB); | NO |
| rs11949931 | NA | NA | NO |
| rs12099669 | NA | Sitting height(UKBB); Fat-free mass(UKBB);Water mass(UKBB) | NO |
| rs12425282 | NA | NA | NO |
| rs12740679 | CAD | NA | YES(CAD) |
| rs12889267 | resting heart rate | Height(UKBB); Fat-free mass(UKBB);Pulse rate(UKBB) | NO |
| rs12928404 | diabetes | Height(UKBB); Fat-free mass(UKBB);Water mass(UKBB);Body mass index(UKBB);Weight(UKBB) | YES(BMI, diabetes) |
| rs12949046 | NA | Height(UKBB); Fat-free mass(UKBB);Water mass(UKBB);Forced vital capacity(UKBB) | NO |
| rs12991919 | NA | Height(UKBB) | NO |
| rs13011633 | NA | Pulse rate(UKBB) | NO |
| rs13029742 | NA | Height(UKBB) | NO |
| rs13105682 | NA | Height(UKBB); Water mass(UKBB);Body mass index(UKBB);Weight(UKBB);Diastolic blood pressure(UKBB) | YES(BMI,hypertension) |
| rs13298297 | glomerular filtration rate | Glomerular filtration rate creatinine(OMID:28452372);fat-free mass(UKBB) | NO |
| rs13399936 | NA | NA | NO |
| rs139603701 | NA | NA | NO |
| rs1418282 | NA | NA | NO |
| rs1440152 | NA | Height(UKBB) | NO |
| rs1480474 | diabetes | Height(UKBB); Fat-free mass(UKBB);Body mass index(PMID:26426971);Weight(UKBB);Hip circumference(UKBB) | YES(BMI,diabetes) |
| rs1556660 | NA | NA | NO |
| rs1558461 | NA | NA | NO |
| rs16910750 | NA | Height(UKBB); Fat-free mass(UKBB);Water mass(UKBB) | NO |
| rs17008195 | NA | NA | NO |
| rs17046434 | NA | Height(UKBB); Fat-free mass(UKBB);Water mass(UKBB) | NO |
| rs17098787 | NA | Height(UKBB); Fat-free mass(UKBB);Water mass(UKBB) | NO |
| rs17368853 | NA | NA | NO |
| rs17536644 | NA | Height(UKBB) | NO |
| rs17631394 | body mass index | Height(UKBB); Fat-free mass(UKBB);Water mass(UKBB);Forced vital capacity(UKBB);Body mass index(UKBB) | YES(BMI) |
| rs185490114 | NA | NA | NO |
| rs1883640 | NA | NA | NO |
| rs188412997 | NA | NA | NO |
| rs1999292 | NA | NA | NO |
| rs2024471 | NA | Fat-free mass(UKBB);Water mass(UKBB);Body mass index(UKBB);Weight(UKBB);Hip circumference(UKBB) | YES(BMI) |
| rs2043962 | NA | NA | NO |
| rs2048719 | NA | NA | NO |
| rs2246005 | NA | Diastolic blood pressure(UKBB) | YES(hypertension) |
| rs2307075 | NA | NA | NO |
| rs2341569 | hip circumference | fat-free mass(UKBB) | NO |
| rs2427574 | NA | NA | NO |
| rs2489364 | NA | NA | NO |
| rs249520 | NA | Height(UKBB); Fat-free mass(UKBB);water mass(UKBB);Hip circumference(UKBB) | NO |
| rs2798631 | NA | Height(UKBB); Fat-free mass(UKBB);Water mass(UKBB); Forced vital capacity(UKBB) | NO |
| rs2894602 | NA | Height(UKBB); Fat-free mass(UKBB);Water mass(UKBB) | NO |
| rs2971154 | NA | Height(UKBB); Fat-free mass(UKBB);Water mass(UKBB) | NO |
| rs3132442 | NA | Height(UKBB); Fat mass(UKBB);Water mass(UKBB);Weight(UKBB);Self-reported hypertension(UKBB) | YES(hypertension) |
| rs34217742 | NA | NA | NO |
| rs34465449 | NA | Height(UKBB);Alcohol intake frequency(UKBB);Systolic blood pressure(UKBB) | YES(drinking, hypertension) |
| rs34824085 | NA | NA | NO |
| rs35166564 | NA | Height(UKBB); Fat mass(UKBB);Water mass(UKBB);Weight(UKBB);Diastolic blood pressure(UKBB) | YES(hypertension) |
| rs35184771 | NA | Height(UKBB);Body mass index(UKBB);Alcohol intake frequency(UKBB);Smoking status: current(UKBB) | YES(BMI,smoking, drinking) |
| rs35809681 | NA | Height(UKBB); Fat mass(UKBB) | NO |
| rs36028059 | NA | NA | NO |
| rs36112366 | body height | Height(UKBB); Fat-free mass(UKBB);Water mass(UKBB);Weight(UKBB) | NO |
| rs3781295 | NA | Atrial fibrillation and flutter(UKBB) | YES(atrial fibrillation) |
| rs3887753 | NA | NA | NO |
| rs398745 | NA | NA | NO |
| rs417614 | NA | NA | NO |
| rs4310973 | vital capacity | Height(UKBB); Fat-free mass(UKBB);Water mass(UKBB);Weight(UKBB) | NO |
| rs4379363 | NA | NA | NO |
| rs4393380 | NA | NA | NO |
| rs4393510 | NA | NA | NO |
| rs4517588 | NA | NA | NO |
| rs4696510 | NA | NA | NO |
| rs4751671 | NA | Height(UKBB) | NO |
| rs4783718 | NA | Height(UKBB); Fat mass(UKBB);Forced vital capacity(UKBB);Body mass index(UKBB);Pulse rate(UKBB) | YES(BMI) |
| rs4795318 | NA | Height(UKBB); Fat-free mass(UKBB);Water mass(UKBB) | NO |
| rs4802848 | NA | NA | NO |
| rs4869851 | NA | NA | NO |
| rs4945185 | NA | NA | NO |
| rs4994327 | NA | NA | NO |
| rs55706579 | NA | NA | NO |
| rs55915134 | NA | Height(UKBB); Fat-free mass(UKBB);Water mass(UKBB) | NO |
| rs56074046 | NA | Height(UKBB); Fat-free mass(UKBB) | NO |
| rs56100703 | NA | Height(UKBB); Fat-free mass(UKBB);Water mass(UKBB) | NO |
| rs56223081 | NA | Height(UKBB); Fat-free mass(UKBB);Water mass(UKBB);Weight(UKBB) | NO |
| rs56403662 | NA | NA | NO |
| rs564340 | NA | Height(UKBB) | NO |
| rs57884925 | NA | NA | NO |
| rs58319813 | NA | NA | NO |
| rs6007580 | NA | NA | NO |
| rs6060355 | body height | Height(UKBB); Fat-free mass(UKBB);Water mass(UKBB);Weight(UKBB) | NO |
| rs6087571 | NA | Height(UKBB);Fat mass(UKBB); Fat-free mass(UKBB);Water mass(UKBB);Weight(UKBB) | NO |
| rs61780429 | NA | Height(UKBB); Fat-free mass(UKBB);Water mass(UKBB);Weight(UKBB); Diastolic blood pressure(UKBB) | YES(hypertension) |
| rs62253602 | NA | Height(UKBB); Body mass index(UKBB);Coronary artery disease(PMID:29212778) | YES(BMI,CAD) |
| rs62346126 | body height | Height(UKBB); Fat-free mass(UKBB);Water mass(UKBB);Weight(UKBB); Diastolic blood pressure(UKBB) | Hypertension |
| rs62621197 | body fat distribution | Height(UKBB); Fat-free mass(UKBB);Water mass(UKBB);Weight(UKBB) | NO |
| rs6573308 | NA | Height(UKBB); Fat-free mass(UKBB);Water mass(UKBB);Weight(UKBB); | NO |
| rs6679313 | NA | Height(UKBB) | NO |
| rs6731993 | NA | Type II diabetes(PMID:28566273) | YES(diabetes) |
| rs6792762 | NA | Fat-free mass(UKBB);Water mass(UKBB);Alcohol intake frequency(UKBB) | YES(drinking) |
| rs6864888 | NA | Height(UKBB);Fat mass(UKBB); Fat-free mass(UKBB);Water mass(UKBB) | NO |
| rs6867409 | NA | fat mass(UKBB),Body mass index(UKBB) | YES(BMI) |
| rs6882168 | NA | Height(UKBB);Fat mass(UKBB); Fat-free mass(UKBB);Water mass(UKBB); | NO |
| rs6902789 | NA | Height(UKBB);Fat mass(UKBB); Fat-free mass(UKBB);Water mass(UKBB);Weight(UKBB) | NO |
| rs7016366 | NA | NA | NO |
| rs701810 | NA | NA | NO |
| rs7084705 | NA | Height(UKBB);Fat mass(UKBB); Fat-free mass(UKBB);Water mass(UKBB) | NO |
| rs72751292 | NA | NA | NO |
| rs7314075 | NA | NA | NO |
| rs75069534 | NA | NA | NO |
| rs7608976 | NA | Height(UKBB); Fat mass(UKBB);Hip circumference(UKBB); Body mass index（UKBB） | BMI |
| rs76310549 | NA | NA | NO |
| rs7633561 | NA | Fat-free mass(UKBB);Water mass(UKBB) | NO |
| rs7636670 | NA | NA | NO |
| rs76895963 | diabetes;LDL;CVDs;systolic blood pressure | Height(UKBB); Fat-free mass(UKBB);Water mass(UKBB);Weight(UKBB);Self-reported diabetes(UKBB) | YES(diabetes,CVDs,hypertension, lipid profile) |
| rs7701967 | NA | Height(UKBB); Fat-free mass(UKBB);Water mass(UKBB) | NO |
| rs7741360 | NA | Height(UKBB); Fat-free mass(UKBB);Water mass(UKBB);Weight(UKBB) | NO |
| rs7766407 | NA | NA | NO |
| rs7790322 | NA | Height(UKBB); Fat-free mass(UKBB);Water mass(UKBB);Weight(UKBB) | NO |
| rs7795051 | NA | NA | NO |
| rs7909837 | NA | Height(UKBB) | NO |
| rs8012800 | NA | NA | NO |
| rs8049014 | NA | Height(UKBB); Water mass(UKBB);Weight(UKBB);Systolic blood pressure(UKBB);Body mass index(UKBB) | YES(hypertension,BMI) |
| rs806292 | NA | NA | NO |
| rs8069671 | NA | Height(UKBB) | NO |
| rs8180765 | NA | NA | NO |
| rs911641 | NA | Height(UKBB); Fat-free mass(UKBB);Water mass(UKBB) | NO |
| rs9284832 | NA | Height(UKBB); Fat-free mass(UKBB);Water mass(UKBB) | NO |
| rs9400284 | NA | Height(UKBB); Fat-free mass(UKBB);Water mass(UKBB);Weight(UKBB) | NO |
| rs945890 | NA | Height(UKBB); Fat-free mass(UKBB);Water mass(UKBB);Weight(UKBB) | NO |
| rs9469813 | NA | Height(UKBB); Fat-free mass(UKBB);Body mass index(UKBB) | BMI |
| rs9894577 | NA | Height(UKBB); Fat-free mass(UKBB);Water mass(UKBB); blood pressure(UKBB) | YES(hypertension) |
| rs9931120 | NA | Fat-free mass(UKBB);Water mass(UKBB) | NO |
| rs9952698 | NA | Height(UKBB); Fat-free mass(UKBB);Water mass(UKBB) | NO |
| rs9987012 | NA | NA | NO |
| rs9991590 | NA | NA | NO |
| rs7034200 | type ii diabetes mellitus | Type II diabetes(PMID:24509480) | YES(diabetes) |

Abbreviations:SNP,single-nucleotide polymorphism. CHR, chromosome. MR,mendelian randomization. UKBB, UK biobank.BMI, body mass index. CAD, coronary artery diseases.

# Supplemental Table 5. Evidence of association (p<5*10-6) of the SNPs used as genetic variants for Mendelian randomization analyses of left hangrip strength with confounders or CVDs in the PhenoScanner and the GWAS catalog.

| SNP | GWAS catalog traits linked to this gene | PhenoScanner traits linked to this gene | Excluded from complementary analysis |
| --- | --- | --- | --- |
| rs10043473 | NA | Job involves heavy manual or physical work(UKBB) | NO |
| rs10176878 | NA | Pulse rate(UKBB) | NO |
| rs10403906 | NA | Height(UKBB);Sleeplessness or insomnia(UKBB) | NO |
| rs10445885 | NA | NA | NO |
| rs10493174 | NA | Height(UKBB) | NO |
| rs10496731 | HDL;body fat percentage | Pulse rate(UKBB);Systolic blood pressure(UKBB);Whole body fat mass(UKBB) | YES(lipid profile, hypertension) |
| rs10753823 | NA | Height(25282103) | NO |
| rs10798482 | NA | Fat-free mass(UKBB);Height(UKBB);Whole body water mass(UKBB) | NO |
| rs10851633 | NA | NA | NO |
| rs10883733 | NA | NA | NO |
| rs10916148 | NA | Fat-free mass(UKBB);Height(UKBB);Whole body water mass(UKBB) | NO |
| rs10988217 | NA | Body mass index males(28892062) | BMI |
| rs11003014 | NA | Height(UKBB);Whole body fat-free mass(UKBB);Whole body water mass(UKBB) | NO |
| rs11067228 | NA | Height(28146470);Fat-free mass right(UKBB) | NO |
| rs11073613 | NA | Height(UKBB) | NO |
| rs11121529 | NA | Asthma(UKBB);Height(UKBB) | NO |
| rs112570672 | BMI-adjusted hip circumference | Height(UKBB);Weight(UKBB);Whole body fat-free mass(UKBB);Whole body water mass(UKBB) | NO |
| rs113602522 | NA | NA | NO |
| rs115771255 | NA | Coronary artery disease(29212778) | YES(CAD) |
| rs11676702 | NA | Height(UKBB);Whole body fat-free mass(UKBB);Whole body water mass(UKBB) | NO |
| rs116825011 | NA | NA | NO |
| rs1177590 | NA | NA | NO |
| rs117844066 | LDL, sleep duration | Self-reported high cholesterol(UKBB) | YES(lipid profile) |
| rs11826567 | NA | Height(UKBB);Whole body fat-free mass(UKBB) | NO |
| rs1205346 | NA | Height(UKBB);Past tobacco smoking(UKBB);Weight(UKBB);Whole body fat-free mass(UKBB);Whole body water mass(UKBB) | YES(smoking) |
| rs12146743 | NA | NA | NO |
| rs12153391 | body height | Height(UKBB) | NO |
| rs1217456 | NA | Basal metabolic rate(UKBB);Pulse rate(UKBB);Whole body fat-free mass(UKBB);Whole body water mass(UKBB) | NO |
| rs12356830 | NA | NA | NO |
| rs12435835 | NA | Height(UKBB);Whole body fat-free mass(UKBB);Whole body water mass(UKBB) | NO |
| rs12889267 | lean body mass | Resting heart rate(27798624);Height(UKBB);Pulse rate(UKBB);Whole body fat-free mass(UKBB);Whole body water mass(UKBB) | NO |
| rs12902985 | NA | Height(UKBB);Whole body fat-free mass(UKBB);Whole body water mass(UKBB) | NO |
| rs12905519 | NA | NA | NO |
| rs13023088 | intelligence | Years of educational attainment(27225129) | NO |
| rs13091492 | NA | Fed-up feelings(UKBB);Guilty feelings(UKBB) | NO |
| rs13150083 | NA | NA | NO |
| rs1317490 | NA | NA | NO |
| rs13213814 | NA | Medication for cholesterol, blood pressure or diabetes: blood pressure medication(UKBB);Sitting height(UKBB) | YES(lipid profile, hypertension） |
| rs13265437 | NA | Height(UKBB) | NO |
| rs13298297 | glomerular filtration rate | Glomerular filtration rate creatinine(28452372);Sitting height(UKBB);Whole body fat-free mass(UKBB) | NO |
| rs13331451 | NA | Height(UKBB);Self-reported hypertension(UKBB);Weight(UKBB);Whole body fat mass(UKBB) | YES(hypertension） |
| rs141615456 | NA | NA | NO |
| rs1418653 | NA | Height(UKBB);Weight(UKBB);Whole body fat-free mass(UKBB);Whole body water mass(UKBB) | NO |
| rs142925250 | NA | NA | NO |
| rs1480474 | type ii diabetes mellitus | Body mass index(26426971);Height(20881960);Whole body fat mass(UKBB);Whole body fat-free mass(UKBB);Whole body water mass (UKBB) | YES(BMI, diabetes) |
| rs16910750 | NA | Height(25282103);Pulse rate(UKBB);Whole body fat-free mass(UKBB);Whole body water mass(UKBB) | NO |
| rs17140345 | NA | NA | NO |
| rs17282763 | NA | NA | NO |
| rs17536644 | NA | NA | NO |
| rs1853927 | NA | Height(UKBB);Whole body fat-free mass(UKBB);Whole body water mass(UKBB) | NO |
| rs185490114 | NA | Rheumatoid arthritis(24390342);Rheumatoid arthritis(24390342) | NO |
| rs1862901 | NA | Height(UKBB);Weight(UKBB);Whole body fat-free mass(UKBB);Whole body water mass(UKBB) | NO |
| rs187673257 | NA | NA | NO |
| rs1884447 | NA | NA | NO |
| rs1918202 | intelligence | Fluid intelligence score(UKBB);Nervous feelings(UKBB) | NO |
| rs1971940 | NA | Height(UKBB) | NO |
| rs2035609 | NA | NA | NO |
| rs2045556 | NA | Height(UKBB);Hip circumference(UKBB);Weight(UKBB);Whole body fat-free mass(UKBB);Whole body water mass(UKBB) | NO |
| rs2163971 | NA | Alcohol intake frequency(UKBB);Body mass index(UKBB);Ever smoked(UKBB);Types of physical activity in last 4 weeks(UKBB);Weight(UKBB);Whole body fat mass(UKBB);Whole body fat-free mass(UKBB);Whole body water mass(UKBB) | YES(BMI, drinking) |
| rs218341 | NA | Height(UKBB);Weight(UKBB);Whole body fat-free mass(UKBB);Whole body water mass(UKBB) | NO |
| rs224390 | NA | Weight(23754948);Total cholesterol(20686565);Height(UKBB);Whole body fat-free mass(UKBB);Whole body water mass(UKBB) | YES(lipid profile) |
| rs2291119 | NA | Body mass index(23754948);Height(25282103);High density lipoprotein(24097068);Triglycerides(24097068) | YES(BMI) |
| rs2355371 | NA | Alcohol intake frequency(UKBB);Body mass index(UKBB);Weight(UKBB);Whole body fat mass(UKBB);Whole body fat-free mass(UKBB);Whole body water mass(UKBB) | YES(drinking) |
| rs2431108 | major depressive disorder | Body mass index(UKBB);Ever depressed for a whole week(UKBB);Whole body fat mass(UKBB);Years of educational attainment(27225129) | BMI |
| rs2565689 | NA | Height(21998595);Weight(UKBB);Whole body fat-free mass(UKBB);Whole body water mass(UKBB) | NO |
| rs2807504 | NA | NA | NO |
| rs28409768 | NA | Heart failure(UKBB);Height(UKBB);Whole body fat-free mass(UKBB);Whole body water mass(UKBB) | NO |
| rs2974438 | NA | Height(20881960);Weight(UKBB);Whole body fat-free mass(UKBB);Whole body water mass(UKBB) | NO |
| rs34159998 | NA | NA | NO |
| rs343957 | NA | Height(UKBB) | NO |
| rs34824085 | NA | NA | NO |
| rs35236379 | NA | NA | NO |
| rs36086419 | NA | NA | NO |
| rs3781295 | NA | Atrial fibrillation and flutter(UKBB);Self-reported atrial fibrillation(UKBB);Years of educational attainment(27225129) | YES(atrial fibrillation) |
| rs3829814 | NA | Forced vital capacity(UKBB);Height(UKBB) | NO |
| rs3850625 | glomerular filtration rate | Glomerular filtration rate creatinine(28452372);Whole body fat-free mass(UKBB);Whole body water mass(UKBB) | NO |
| rs4128846 | NA | Height(UKBB);Whole body fat-free mass(UKBB);Whole body water mass(UKBB) | NO |
| rs417591 | NA | Sleep duration(UKBB) | NO |
| rs4310973 | vital capacity | Height(UKBB);Weight(UKBB);Whole body fat mass(UKBB);Whole body fat-free mass(UKBB);Whole body water mass(UKBB) | NO |
| rs4393380 | NA | NA | NO |
| rs4705885 | NA | Asthma(UKBB);Height(UKBB);Whole body fat-free mass(UKBB);Whole body water mass(UKBB) | NO |
| rs4783718 | NA | Body mass index(UKBB);Height(UKBB);Pulse rate(UKBB);Whole body fat mass(UKBB) | YES(BMI) |
| rs4784328 | NA | Body mass index(UKBB) | YES(BMI) |
| rs4795318 | NA | Height(UKBB);Whole body fat-free mass(UKBB);Whole body water mass(UKBB) | NO |
| rs4797782 | NA | NA | NO |
| rs4802848 | NA | NA | NO |
| rs4903247 | NA | Worrier or anxious feelings(UKBB) | NO |
| rs491347 | NA | Height(UKBB);Weight(UKBB);Whole body fat-free mass(UKBB);Whole body water mass(UKBB) | NO |
| rs4994327 | NA | NA | NO |
| rs553108 | Calcium channel blocker use measurement | Diastolic blood pressure(UKBB);Height(UKBB);Self-reported high cholesterol(UKBB);Self-reported hypertension(UKBB);Self-reported rheumatoid arthritis(UKBB);Self-reported systemic lupus erythematosis (UKBB);Systolic blood pressure(UKBB);Weight(UKBB);Whole body fat mass(UKBB);Whole body fat-free mass(UKBB);Whole body water mass(UKBB) | YES(hypertension,lipid profile) |
| rs56100703 | NA | Height(UKBB);Whole body fat-free mass(UKBB);Whole body water mass(UKBB) | NO |
| rs56402185 | NA | Height(UKBB);Whole body fat-free mass(UKBB);Whole body water mass(UKBB) | NO |
| rs58319813 | NA | NA | NO |
| rs599550 | systolic blood pressure;depression | Body mass index(UKBB);Whole body water mass(UKBB);Depressive symptoms(27089181);Neuroticism(29292387) | YES(BMI, hypertension) |
| rs60750824 | platelet count | NA | NO |
| rs61818099 | NA | NA | NO |
| rs62253602 | NA | Body mass index(UKBB);Height(UKBB);Coronary artery disease(29212778) | YES(CAD,BMI) |
| rs62332075 | NA | NA | NO |
| rs62346126 | body height | Height(UKBB);Weight(UKBB);Whole body fat mass(UKBB);Whole body fat-free mass(UKBB);Whole body water mass(UKBB) | NO |
| rs6433478 | physical activity | NA | NO |
| rs6503398 | NA | Height(UKBB) | NO |
| rs6592728 | NA | NA | NO |
| rs6731993 | NA | Type II diabetes(28566273);Illnesses of siblings: diabetes(UKBB);Rheumatoid arthritis(24390342) | YES(diabetes) |
| rs6882168 | NA | Height(UKBB);Whole body fat-free mass(UKBB);Whole body water mass(UKBB) | NO |
| rs701810 | NA | NA | NO |
| rs71351952 | NA | Body mass index(UKBB);Height(UKBB);Whole body fat-free mass(UKBB);Whole body water mass(UKBB) | YES(BMI) |
| rs71528371 | NA | Height(UKBB);Weight(UKBB);Whole body fat-free mass(UKBB);Whole body water mass(UKBB) | NO |
| rs7166220 | NA | Height(UKBB);Whole body fat-free mass(UKBB);Whole body water mass(UKBB) | NO |
| rs7174985 | NA | Body mass index(UKBB);Height(UKBB);Peak expiratory flow(UKBB) | YES(BMI) |
| rs7249081 | lean body mass | Height(UKBB);Hip circumference(UKBB);Weight(UKBB);Whole body fat-free mass(UKBB);Whole body water mass(UKBB) | NO |
| rs72686716 | NA | Body mass index(UKBB);Fluid intelligence score(UKBB);Height(UKBB) | BMI |
| rs72845891 | NA | Height(UKBB);Weight(UKBB);Whole body fat-free mass(UKBB);Whole body water mass(UKBB) | NO |
| rs7301953 | NA | High density lipoprotein(24097068);Triglycerides(24097068);Adiponectin levels(22479202);Body fat percentage(UKBB);Whole body fat mass(UKBB) | YES(lipid profile) |
| rs75069534 | NA | NA | NO |
| rs75659664 | NA | NA | NO |
| rs7576689 | NA | Sitting height(UKBB) | NO |
| rs7608976 | NA | Body mass index(UKBB);Height(UKBB);Whole body fat mass(UKBB) | BMI |
| rs76895963 | type ii diabetes mellitus;body mass index;total cholesterol measurement;cardiovascular disease | Pulse pressure(28135244);Body mass index(UKBB);Diabetes diagnosed by doctor(UKBB);Height(UKBB);Self-reported diabetes(UKBB);Self-reported type 2 diabetes(UKBB);Weight(UKBB);Whole body fat mass(UKBB);Whole body fat-free mass(UKBB);Whole body water mass(UKBB) | YES(diabetes,lipid profile,BMI) |
| rs7756651 | NA | Height(UKBB);Weight(UKBB);Whole body fat-free mass(UKBB);Whole body water mass(UKBB) | NO |
| rs7761910 | NA | Height(UKBB);Whole body fat-free mass(UKBB);Whole body water mass(UKBB) | NO |
| rs7795051 | NA | NA | NO |
| rs78743523 | NA | NA | NO |
| rs79177013 | NA | Height(UKBB);Weight(UKBB);Whole body fat mass(UKBB);Whole body fat-free mass(UKBB);Whole body water mass(UKBB) | NO |
| rs8023263 | NA | Height(UKBB);Whole body fat-free mass(UKBB);Whole body water mass(UKBB) | NO |
| rs8056535 | NA | Alcohol intake frequency(UKBB);Body mass index(UKBB);Self-reported hypertension(UKBB);Weight(UKBB);Whole body fat mass(UKBB);Whole body fat-free mass(UKBB);Whole body water mass(UKBB);Schizophrenia(25056061) | YES(drinking, BMI, hypertension) |
| rs8069671 | NA | Height(UKBB) | NO |
| rs8101782 | NA | Height(UKBB) | NO |
| rs8138982 | NA | NA | NO |
| rs8180765 | NA | NA | NO |
| rs833520 | NA | Height(UKBB);Sitting height(UKBB);Whole body fat-free mass(UKBB);Whole body water mass(UKBB) | NO |
| rs889292 | NA | NA | NO |
| rs9284832 | NA | Height(UKBB);Whole body fat-free mass(UKBB);Whole body water mass(UKBB) | NO |
| rs9396861 | NA | NA | NO |
| rs9436117 | NA | NA | NO |
| rs945890 | NA | Height(25282103);Weight(UKBB);Whole body fat-free mass(UKBB);Whole body water mass(UKBB) | NO |
| rs9987012 | NA | NA | NO |

Abbreviations:SNP,single-nucleotide polymorphism. CHR, chromosome. MR,mendelian randomization. UKBB, UK biobank.BMI, body mass index. CAD, coronary artery diseases.

# Supplemental Table 6. Heterogeneity for the Mendelian randomization analysis.

| Exposures | Outcomes | SNP selection* | Cochran's Q | Degrees of Freedom | P-value |
| --- | --- | --- | --- | --- | --- |
| Right handgrip strength | Coronary artery disease | All | 271.716 | 159 | <0.001 |
|  |  | Removed | 183.165 | 127 | <0.001 |
|  | Myocardial infarction | All | 267.135 | 159 | <0.001 |
|  |  | Removed | 181.341 | 127 | <0.001 |
|  | Atrial fibrillation | All | 498.232 | 159 | <0.001 |
|  |  | Removed | 306.453 | 126 | <0.001 |
|  | Heart failure | All | 232.168 | 159 | <0.001 |
|  |  | Removed | 154.219 | 126 | 0.044 |
|  | Hypertension | All | 221.019 | 159 | <0.001 |
|  |  | Removed | 173.541 | 127 | 0.004 |
|  | Ischemic stroke | All | 202.054 | 159 | 0.012 |
|  |  | Removed | 156.368 | 127 | 0.039 |
|  | Cardioembolic stroke | All | 179.417 | 159 | 0.128 |
|  |  | Removed | 144.633 | 127 | 0.136 |
|  | Large artery stroke | All | 181.493 | 159 | 0.107 |
|  |  | Removed | 128.598 | 127 | 0.444 |
|  | Small vessel stroke | All | 159.193 | 159 | 0.481 |
|  |  | Removed | 127.089 | 127 | 0.481 |
| Left handgrip strength | Coronary artery disease | All | 225.759 | 135 | <0.001 |
|  |  | Removed | 169.272 | 108 | <0.001 |
|  | Myocardial infarction | All | 210.395 | 135 | <0.001 |
|  |  | Removed | 154.363 | 108 | <0.001 |
|  | Atrial fibrillation | All | 496.190 | 135 | <0.001 |
|  |  | Removed | 346.429 | 108 | <0.001 |
|  | Heart failure | All | 160.985 | 135 | 0.063 |
|  |  | Removed | 118.768 | 108 | 0.225 |
|  | Hypertension | All | 202.129 | 135 | <0.001 |
|  |  | Removed | 154.410 | 108 | 0.002 |
|  | Ischemic stroke | All | 142.737 | 135 | 0.308 |
|  |  | Removed | 105.967 | 108 | 0.537 |
|  | Cardioembolic stroke | All | 127.232 | 135 | 0.671 |
|  |  | Removed | 87.107 | 108 | 0.930 |
|  | Large artery stroke | All | 138.611 | 135 | 0.398 |
|  |  | Removed | 94.791 | 108 | 0.814 |
|  | Small vessel stroke | All | 151.1528 | 135 | 0.162 |
|  |  | Removed | 131.244 | 108 | 0.064 |

Abbreviations:SNP, Single-nucleotide polymorphis.

* ‘All’ represents analyses with all selected SNPs, ‘Removed’ represents analyses after removing SNPs associated with relevant confounders or CVDs.

# Supplemental Table 7. MR-Egger pleiotropy test for all SNPS.

| Outcomes | SNPs | MR-Egger | | MR-Egger intercept | |  |
| --- | --- | --- | --- | --- | --- | --- |
|  |  | OR(95%CI) | P-valve | OR(95%CI) | P-valve | I^2^_GX_ |
| Right handgrip strength |  |  |  |  |  |  |
| Coronary artery disease | 160 | 0.888(0.816-0.966) | 0.006 | 1.009(0.999-1.020) | 0.079 | 0.97160 |
| Myocardial infarction | 160 | 0.866(0.790-0.949) | 0.002 | 1.013(1.001-1.024) | 0.027 | 0.97161 |
| Atrial fibrillation | 160 | 0.913(0.846-0.986) | 0.020 | 1.007(0.998-1.017) | 0.133 | 0.97164 |
| Heart failure | 160 | 0.992(0.932-1.056) | 0.796 | 1.001(0.994-1.009) | 0.713 | 0.97166 |
| Hypertension | 160 | 0.983(0.857-1.128) | 0.806 | 1.000(0.984-1.017) | 0.969 | 0.97167 |
| Ischemic stroke | 160 | 0.959(0.890-1.034) | 0.276 | 1.005(0.995-1.014) | 0.321 | 0.97166 |
| Cardioembolic stroke | 160 | 0.990(0.862-1.137) | 0.886 | 1.001(0.984-1.018) | 0.894 | 0.97165 |
| Large artery stroke | 160 | 0.859(0.718-1.028) | 0.097 | 1.014(0.992-1.036) | 0.216 | 0.97166 |
| Small vessel stroke | 160 | 1.217(1.044-1.420) | 0.012 | 0.978(0.959-0.996) | 0.018 | 0.97165 |
| Left handgrip strength |  |  |  |  |  |  |
| Coronary artery disease | 136 | 0.948(0.858-1.047) | 0.292 | 1.001(0.989-1.014) | 0.833 | 0.97122 |
| Myocardial infarction | 136 | 0.944(0.849-1.050) | 0.287 | 1.002(0.989-1.015) | 0.743 | 0.97123 |
| Atrial fibrillation | 136 | 0.895(0.808-0.992) | 0.034 | 1.011(0.998-1.024) | 0.085 | 0.97122 |
| Heart failure | 136 | 1.013(0.946-1.085) | 0.708 | 0.999(0.991-1.007) | 0.792 | 0.97121 |
| Hypertension | 136 | 1.043(0.891-1.221) | 0.602 | 0.990(0.971-1.010) | 0.326 | 0.97131 |
| Ischemic stroke | 136 | 1.010(0.927-1.099) | 0.822 | 0.998(0.988-1.009) | 0.742 | 0.97126 |
| Cardioembolic stroke | 136 | 1.102(0.936-1.298) | 0.242 | 0.990(0.971-1.010) | 0.311 | 0.97127 |
| Large artery stroke | 136 | 0.950(0.767-1.176) | 0.636 | 1.004(0.979-1.031) | 0.737 | 0.97128 |
| Small vessel stroke | 136 | 1.013(0.827-1.241) | 0.901 | 0.995(0.971-1.020) | 0.715 | 0.97127 |

Abbreviations:SNPs, Single-nucleotide polymorphiss.

# Supplemental Table 8. MR-Egger pleiotropy test after removing SNPs associated with confounders or CVDs.

| Outcomes | SNPs | MR-Egger | | MR-Egger intercept | |  |
| --- | --- | --- | --- | --- | --- | --- |
|  |  | OR(95%CI) | P-valve | OR(95%CI) | P-valve | Igx |
| Right handgrip strength |  |  |  |  |  |  |
| Coronary artery disease | 128 | 0.901(0.828-0.981) | 0.016 | 1.008(0.997-1.018) | 0.151 | 0.97129 |
| Myocardial infarction | 128 | 0.88(0.802-0.965) | 0.007 | 1.011(0.999-1.022) | 0.063 | 0.97131 |
| Atrial fibrillation | 127 | 0.942(0.874-1.016) | 0.120 | 1.003(0.994-1.012) | 0.538 | 0.97131 |
| Heart failure | 127 | 0.986(0.926-1.051) | 0.667 | 1.002(0.994-1.01) | 0.622 | 0.97135 |
| Hypertension | 128 | 0.964(0.833-1.115) | 0.621 | 1.002(0.984-1.02) | 0.833 | 0.97131 |
| Ischemic stroke | 128 | 0.962(0.888-1.043) | 0.350 | 1.004(0.994-1.014) | 0.391 | 0.97136 |
| Cardioembolic stroke | 128 | 0.977(0.84-1.137) | 0.766 | 1.001(0.983-1.02) | 0.883 | 0.97134 |
| Large artery stroke | 128 | 0.85(0.708-1.021) | 0.082 | 1.014(0.991-1.037) | 0.227 | 0.97135 |
| Small vessel stroke | 128 | 1.257(1.063-1.485) | 0.007 | 0.974(0.954-0.994) | 0.012 | 0.97135 |
| Left handgrip strength |  |  |  |  |  |  |
| Coronary artery disease | 109 | 0.957(0.859-1.067) | 0.429 | 1.001(0.988-1.014) | 0.921 | 0.97090 |
| Myocardial infarction | 109 | 0.974(0.869-1.092) | 0.655 | 0.999(0.985-1.013) | 0.845 | 0.97091 |
| Atrial fibrillation | 109 | 0.932(0.835-1.04) | 0.208 | 1.006(0.993-1.019) | 0.382 | 0.97087 |
| Heart failure | 109 | 1.027(0.953-1.106) | 0.483 | 0.997(0.988-1.006) | 0.525 | 0.97090 |
| Hypertension | 109 | 1.046(0.88-1.242) | 0.613 | 0.989(0.969-1.011) | 0.324 | 0.97090 |
| Ischemic stroke | 109 | 1.014(0.924-1.112) | 0.770 | 0.998(0.986-1.009) | 0.664 | 0.97091 |
| Cardioembolic stroke | 109 | 1.051(0.875-1.263) | 0.594 | 0.995(0.973-1.017) | 0.666 | 0.97092 |
| Large artery stroke | 109 | 0.979(0.773-1.241) | 0.862 | 1(0.972-1.029) | 1.000 | 0.97093 |
| Small vessel stroke | 109 | 1.014(0.798-1.288) | 0.910 | 0.995(0.966-1.024) | 0.710 | 0.97092 |

Abbreviations:SNPs, Single-nucleotide polymorphiss.

# Supplemental Table 9.SNPs excluded from the outlier corrected MR-PRESSO analyses between handgrip strength and cardiovascular diseases.

| Exposure | Outcome | SNP selection* | OutlierSNP | RSSobs | P-value |
| --- | --- | --- | --- | --- | --- |
| Right handgrip strength | | | | | |
|  | Coronary artery diseases | All | rs116922558 | 0.025379132 | 0.016 |
|  |  |  | rs4751671 | 0.00103292 | 0.016 |
|  |  |  | rs62253602 | 0.001555246 | <0.016 |
|  |  | Removed | NA | NA | NA |
|  | Myocardial infarction | All | rs116922558 | 0.027808 | <0.016 |
|  |  |  | rs2246005 | 0.001990253 | 0.032 |
|  |  | Removed | NA | NA | NA |
|  | Atrial fibrillation | All | rs13011633 | 0.004970823 | 0.032 |
|  |  |  | rs2489364 | 0.000675695 | 0.032 |
|  |  |  | rs34465449 | 0.001303777 | <0.016 |
|  |  |  | rs3781295 | 0.002849795 | <0.016 |
|  |  |  | rs61780429 | 0.001019894 | 0.016 |
|  |  |  | rs76895963 | 0.02024403 | <0.016 |
|  |  |  | rs7790322 | 0.000615873 | 0.016 |
|  |  | Removed | rs13011633 | 0.005148794 | 0.0387 |
|  |  |  | rs2489364 | 0.00065739 | 0.0387 |
|  | Heart failure | All | NA | NA | NA |
|  |  | Removed | NA | NA | NA |
|  | Hypertension | All | NA | NA | NA |
|  |  | Removed | NA | NA | NA |
|  | Ischemic stroke | All | NA | NA | NA |
|  |  | Removed | NA | NA | NA |
|  | Cardioembolic stroke | All | NA | NA | NA |
|  |  | Removed | NA | NA | NA |
|  | Large artery stroke | All | NA | NA | NA |
|  |  | Removed | NA | NA | NA |
|  | Small vessel stroke | All | NA | NA | NA |
|  |  | Removed | NA | NA | NA |
| Left handgrip strength | | | | | |
|  | Coronary artery diseases | All | rs62253602 | 0.001548864 | <0.0136 |
|  |  | Removed | NA | NA | NA |
|  | Myocardial infarction | All | rs7301953 | 0.001645172 | 0.0272 |
|  |  | Removed | NA | NA | NA |
|  | Atrial fibrillation | All | rs10753823 | 0.001349125 | <0.0136 |
|  |  |  | rs1217456 | 0.003130455 | <0.0136 |
|  |  |  | rs1418653 | 0.000710038 | 0.0408 |
|  |  |  | rs16910750 | 0.001212197 | <0.0136 |
|  |  |  | rs3781295 | 0.002716857 | <0.0136 |
|  |  |  | rs6433478 | 0.0007681 | 0.0136 |
|  |  |  | rs7301953 | 0.001091458 | <0.0136 |
|  |  |  | rs76895963 | 0.021821789 | <0.0136 |
|  |  |  | rs9396861 | 0.000992381 | <0.0136 |
|  |  | Removed | rs10753823 | 0.001356424 | <0.0109 |
|  |  |  | rs1217456 | 0.003139236 | <0.0109 |
|  |  |  | rs1418653 | 0.000714025 | 0.0218 |
|  |  |  | rs16910750 | 0.001219484 | <0.0109 |
|  |  |  | rs6433478 | 0.000772398 | <0.0109 |
|  |  |  | rs9396861 | 0.000995297 | <0.0109 |
|  | Heart failure | All | NA | NA | NA |
|  |  | Removed | NA | NA | NA |
|  | Hypertension | All | NA | NA | NA |
|  |  | Removed | NA | NA | NA |
|  | Ischemic stroke | All | NA | NA | NA |
|  |  | Removed | NA | NA | NA |
|  | Cardioembolic stroke | All | NA | NA | NA |
|  |  | Removed | NA | NA | NA |
|  | Large artery stroke | Removed | NA | NA | NA |
|  | Small vessel stroke | All | NA | NA | NA |
|  |  | Removed | NA | NA | NA |

Abbreviations:SNP, Single-nucleotide polymorphis.

* ‘All’ represents analyses with all selected SNPs, ‘Removed’ represents analyses after removing SNPs associated with relevant confounders or CVDs.

# Supplemental Figure 1.The diagram of two-sample MR analysis.


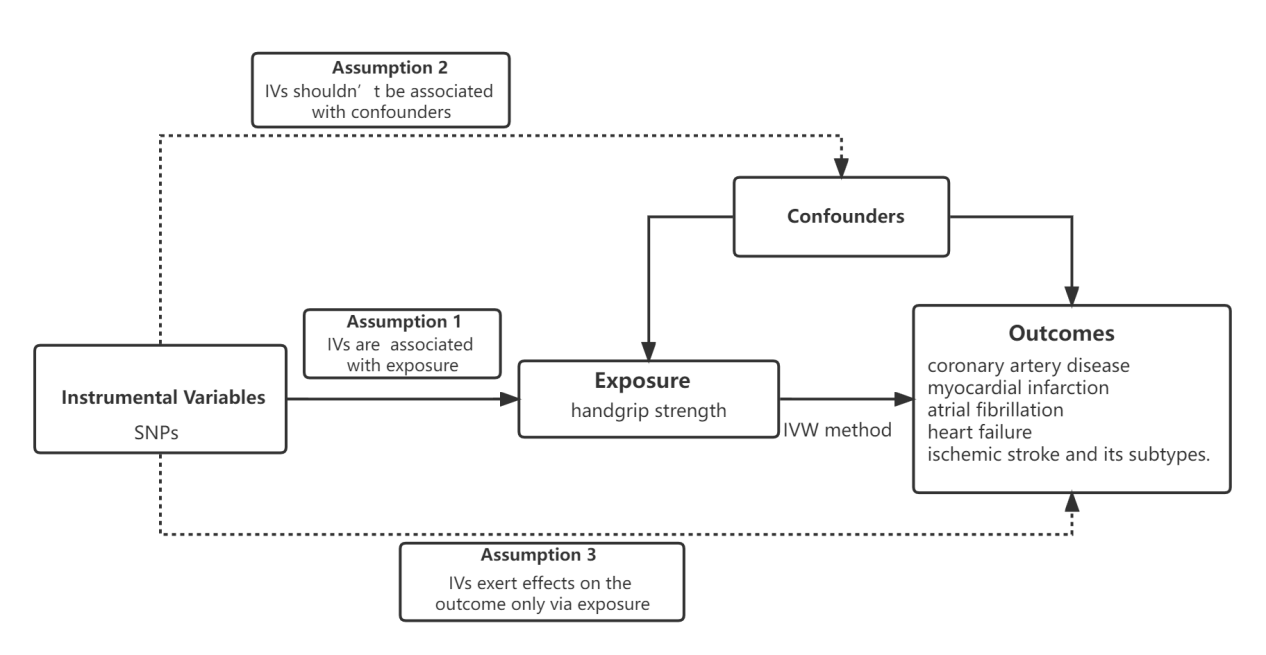


IVs:instrumental variables;IVW:inverse variance-weighted;SNP:single-nucleotide polymorphisms.

#
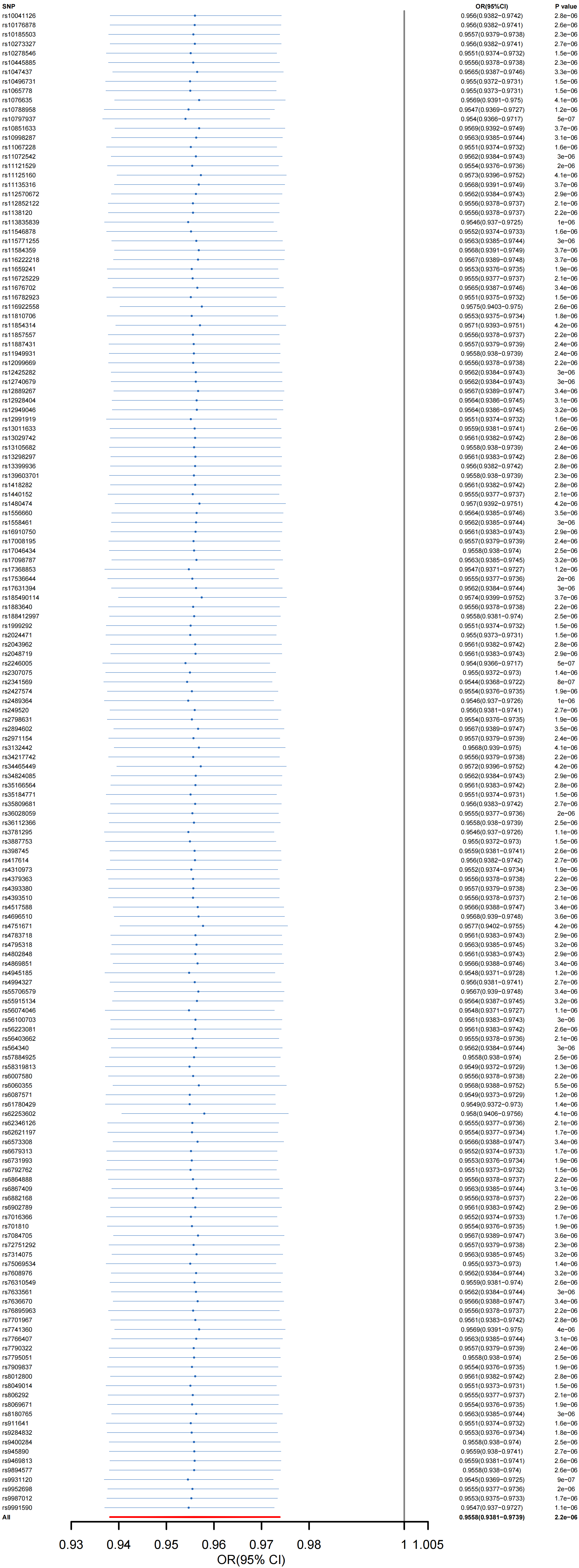
Supplemental Figure 2.MR Leave one out analyses for right handgrip strength on coronary artery disease.


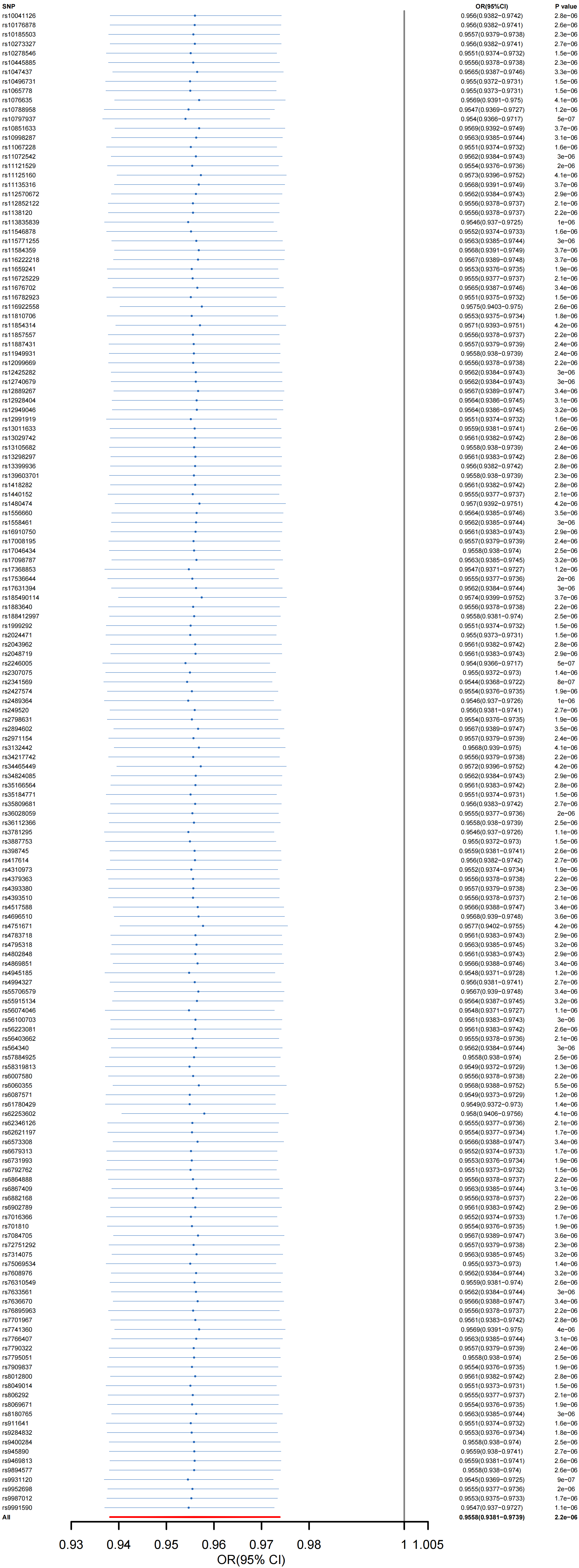


#
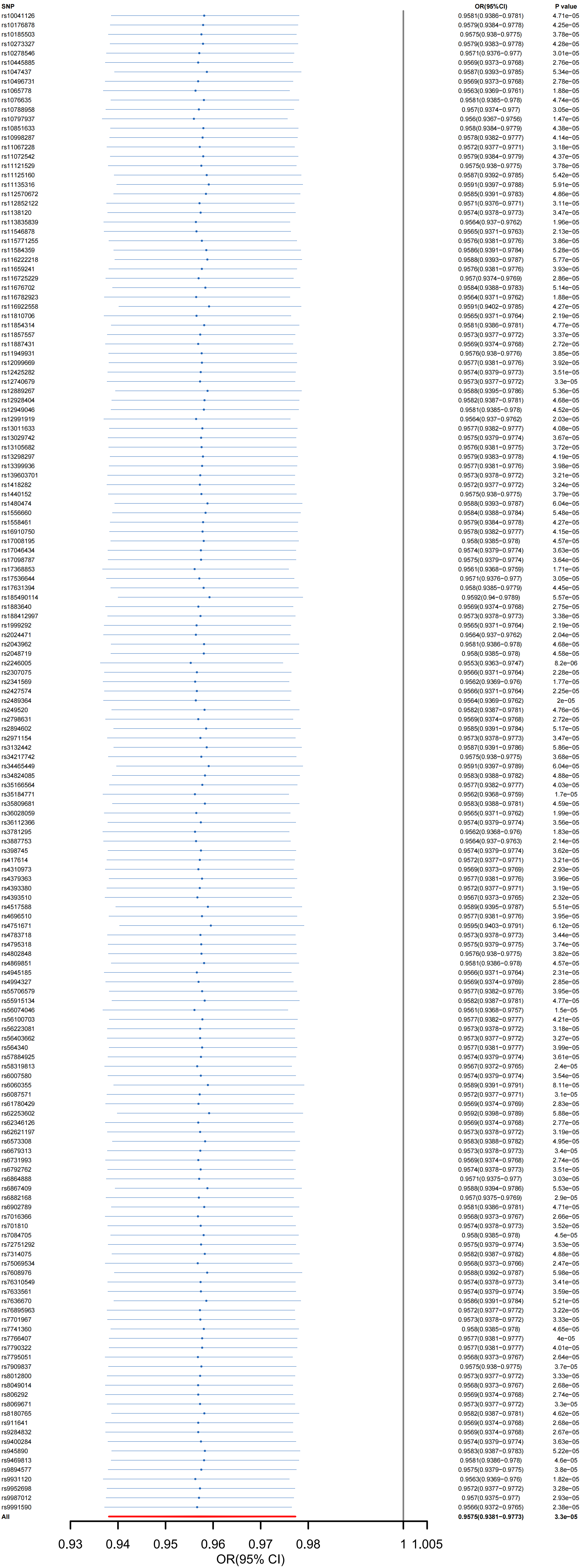
Supplemental Figure 3.MR Leave one out analyses for right handgrip strength on myocardial infarction.


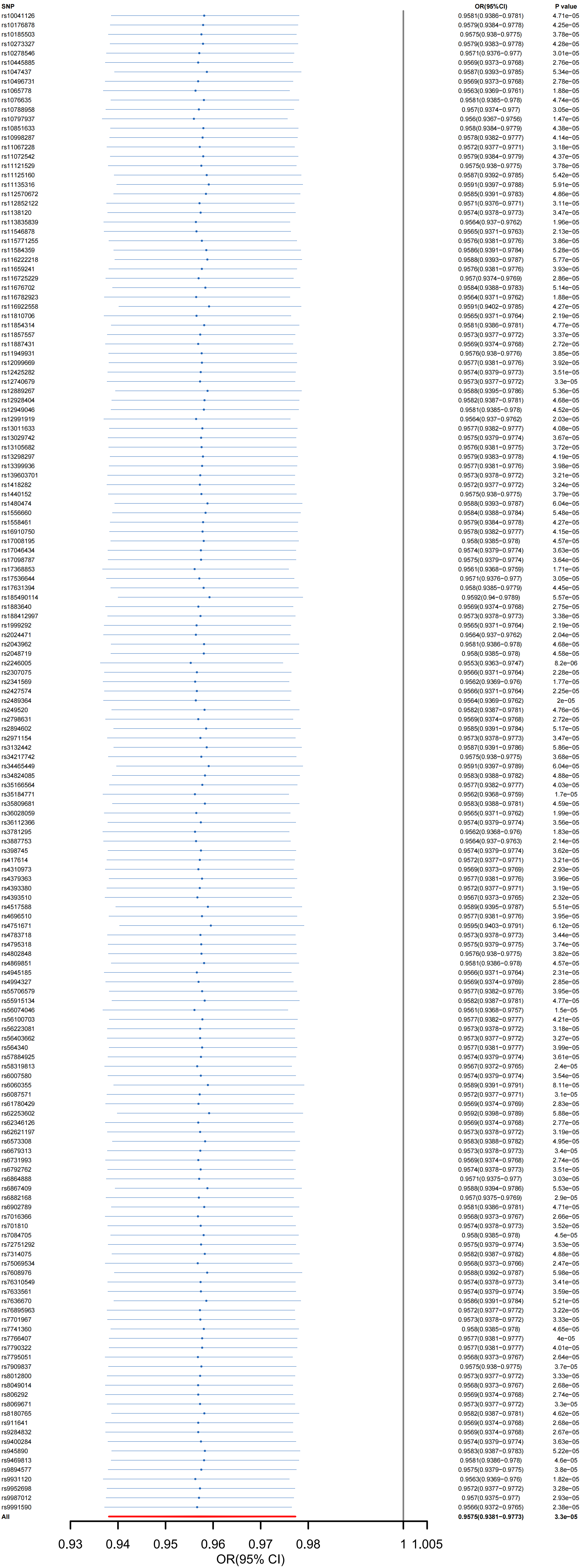


# Supplemental Figure 4.MR Leave one out analyses for right handgrip strength on atrial fibrillation.


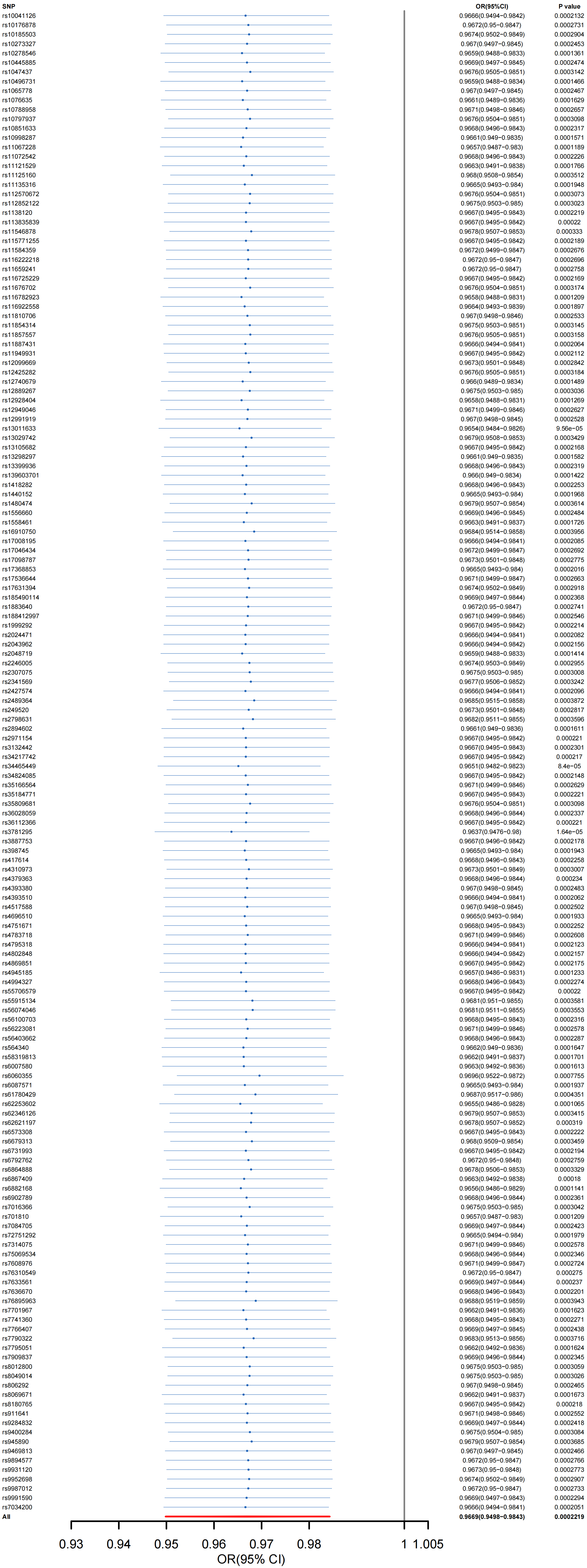


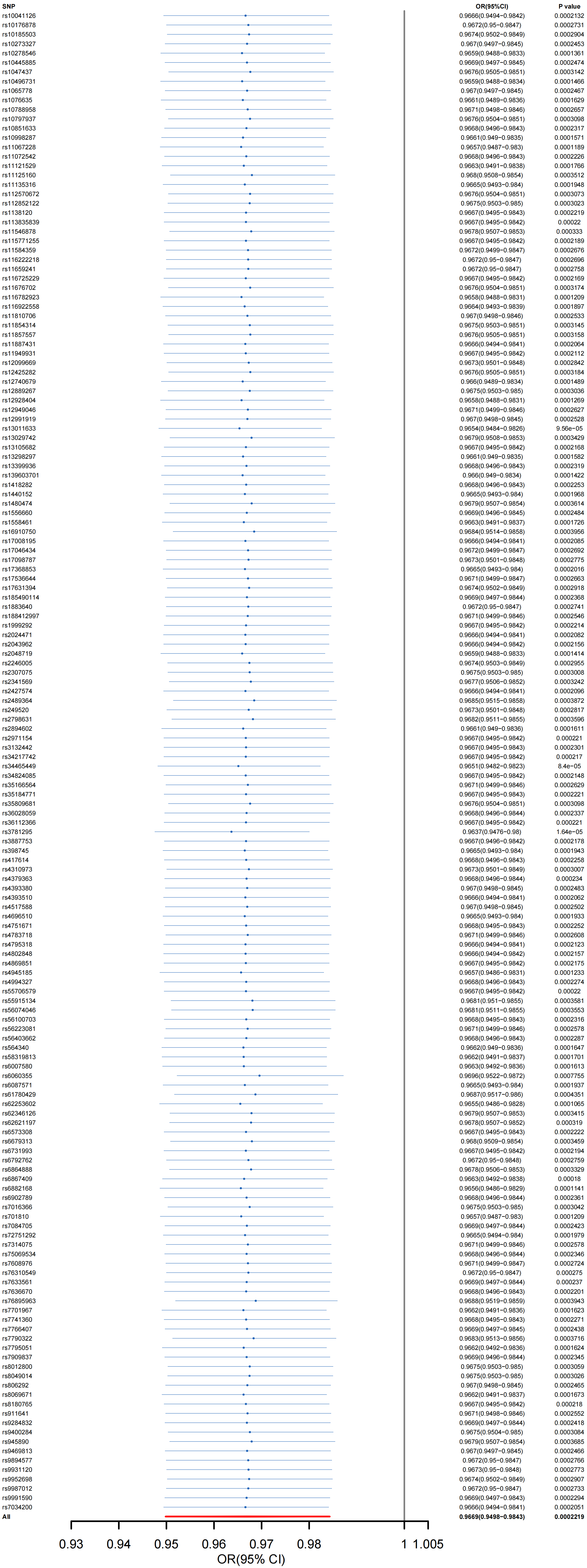


# Supplemental Figure 5.MR Leave one out analyses for left handgrip strength on coronary artery disease.


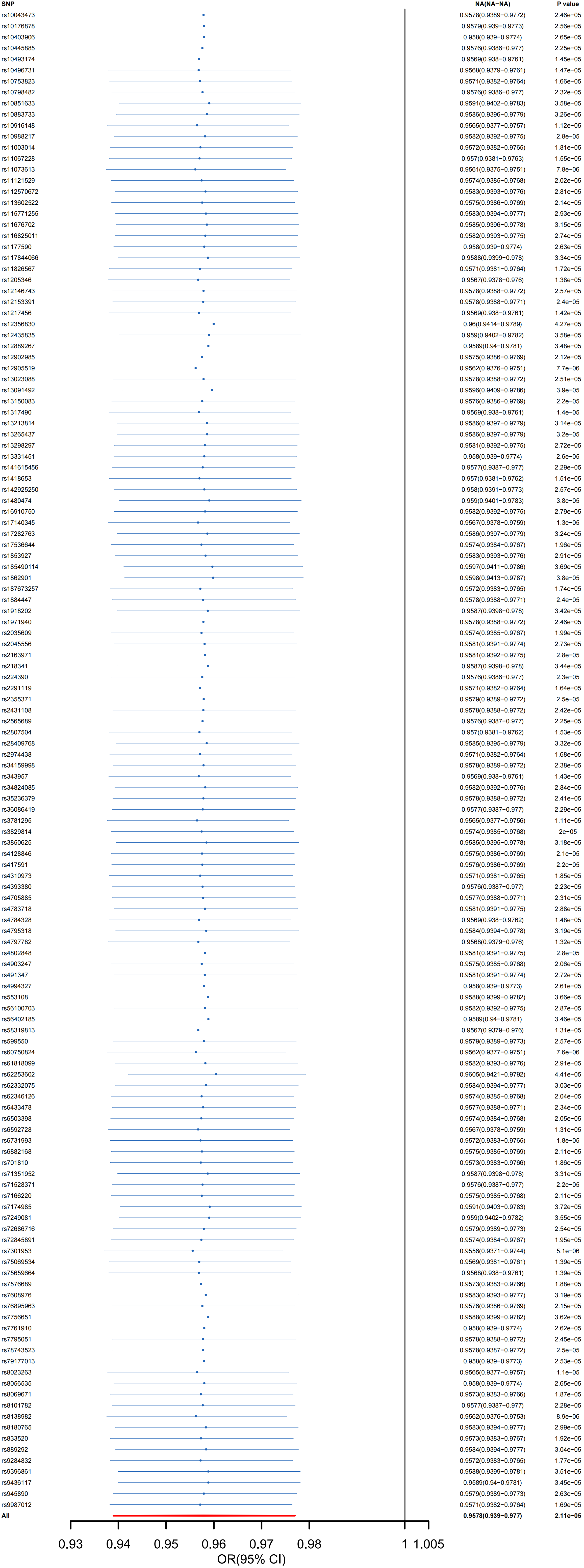


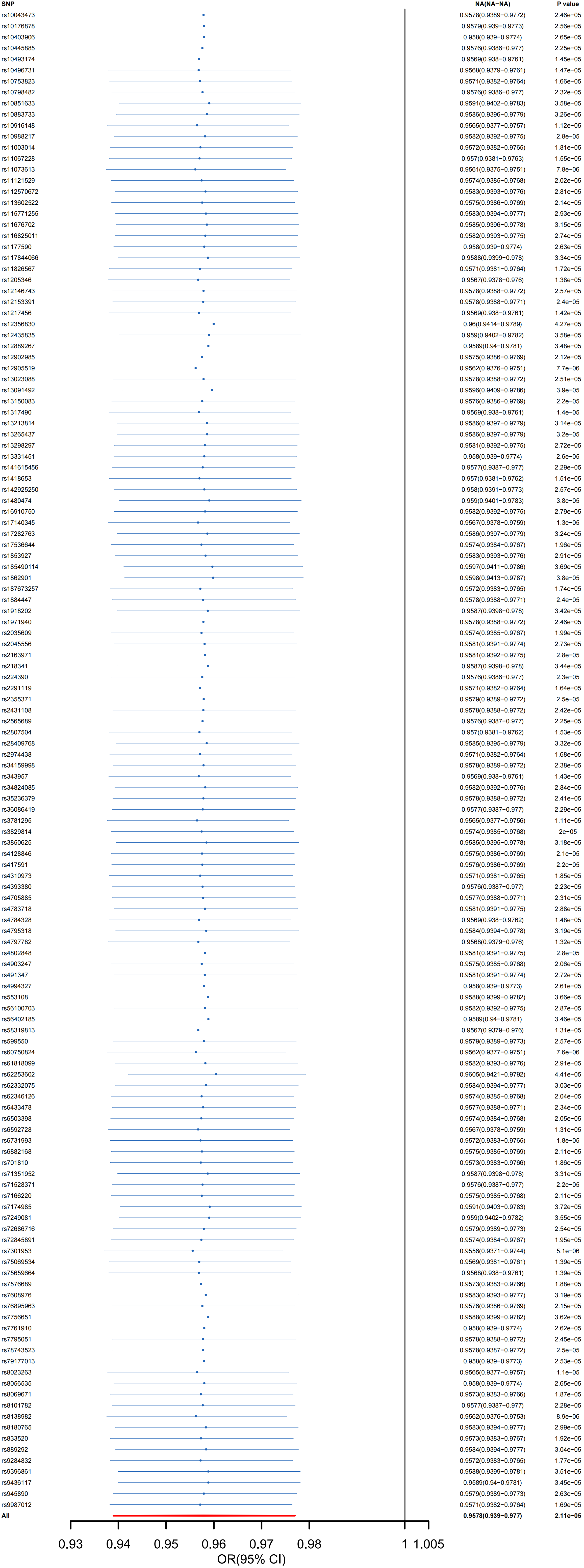


#
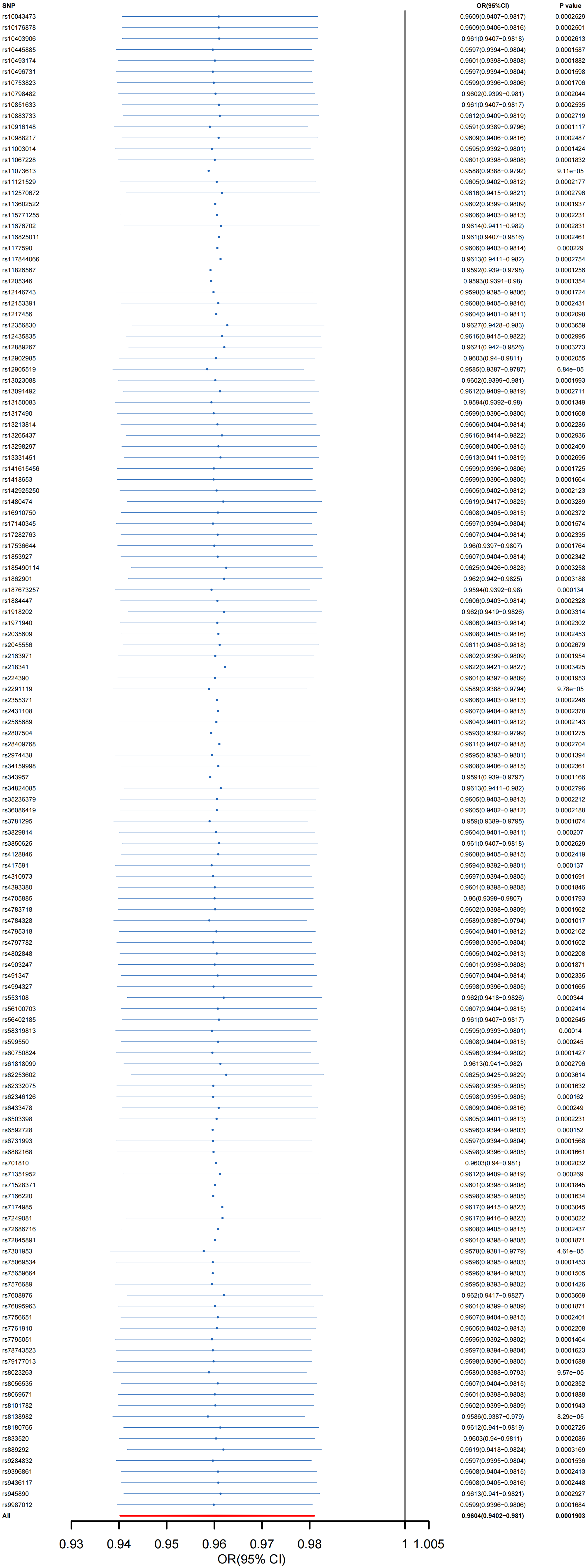
Supplemental Figure 6.MR Leave one out analyses for left handgrip strength on myocardial infarction.


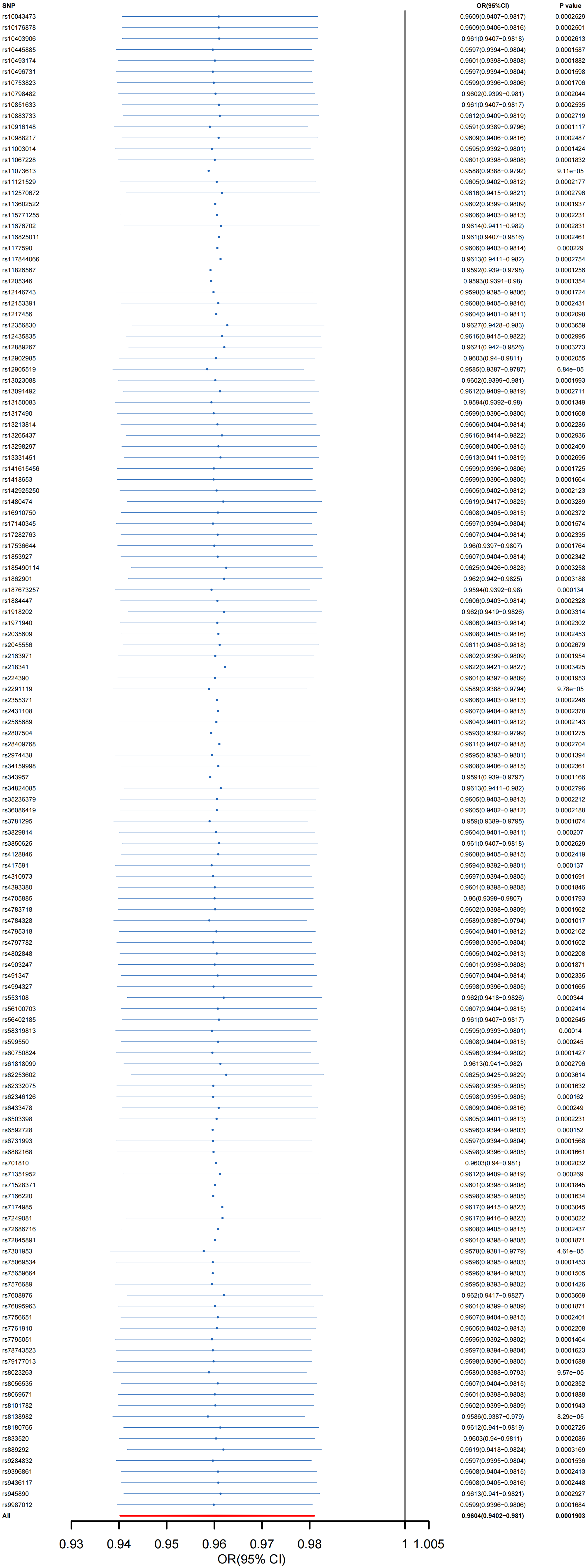


# Supplemental Figure 7.MR Leave one out analyses for left handgrip strength on atrial fibrillation.


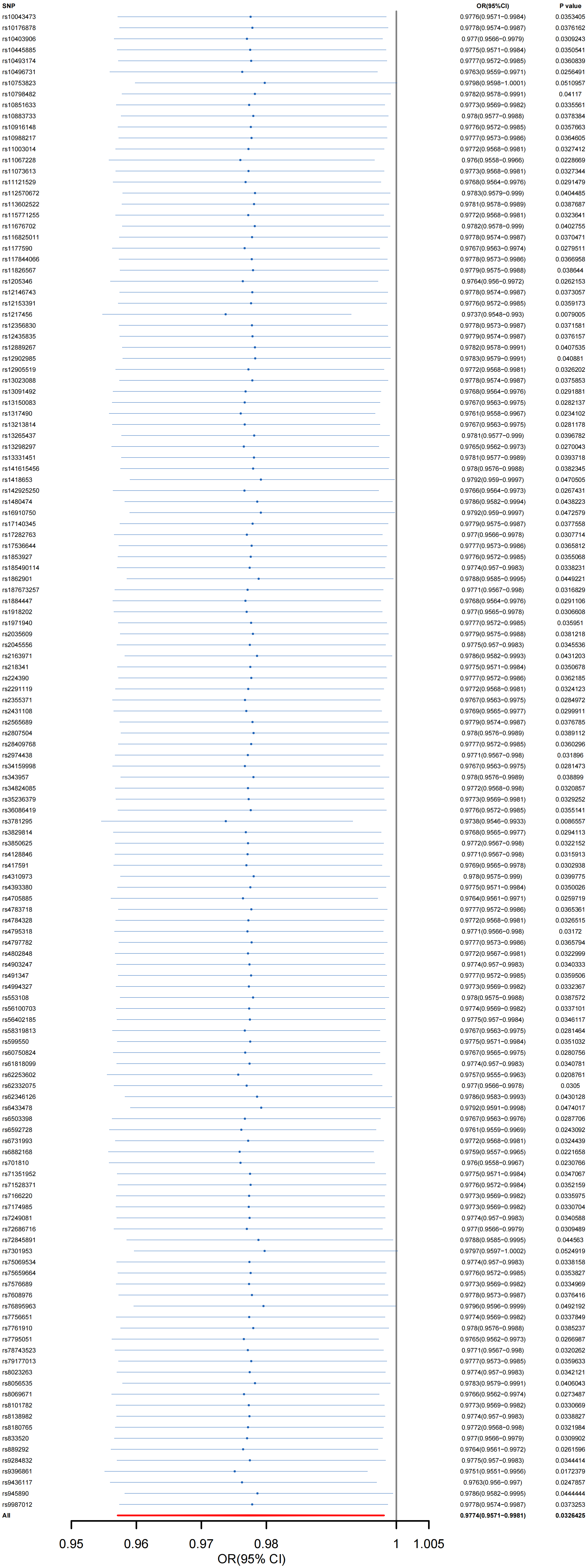


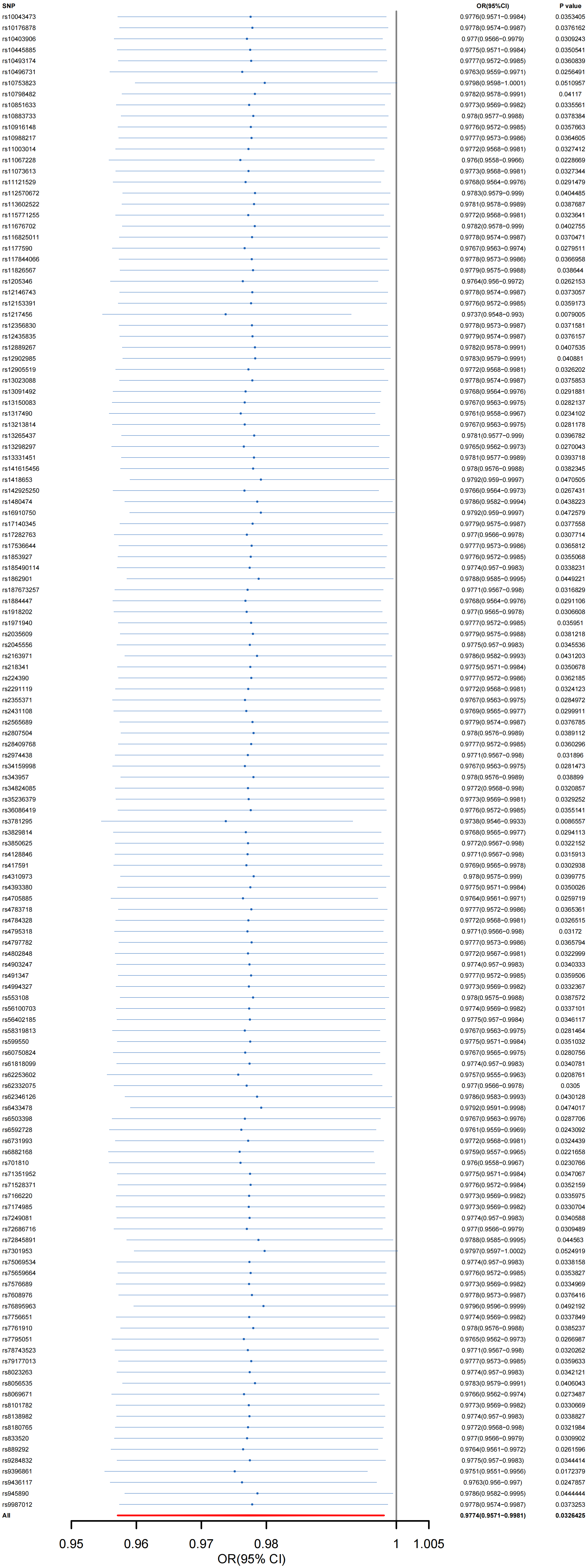


# Supplemental Figure 8.A scatter plot for the causal association of right handgrip strength with outcomes.


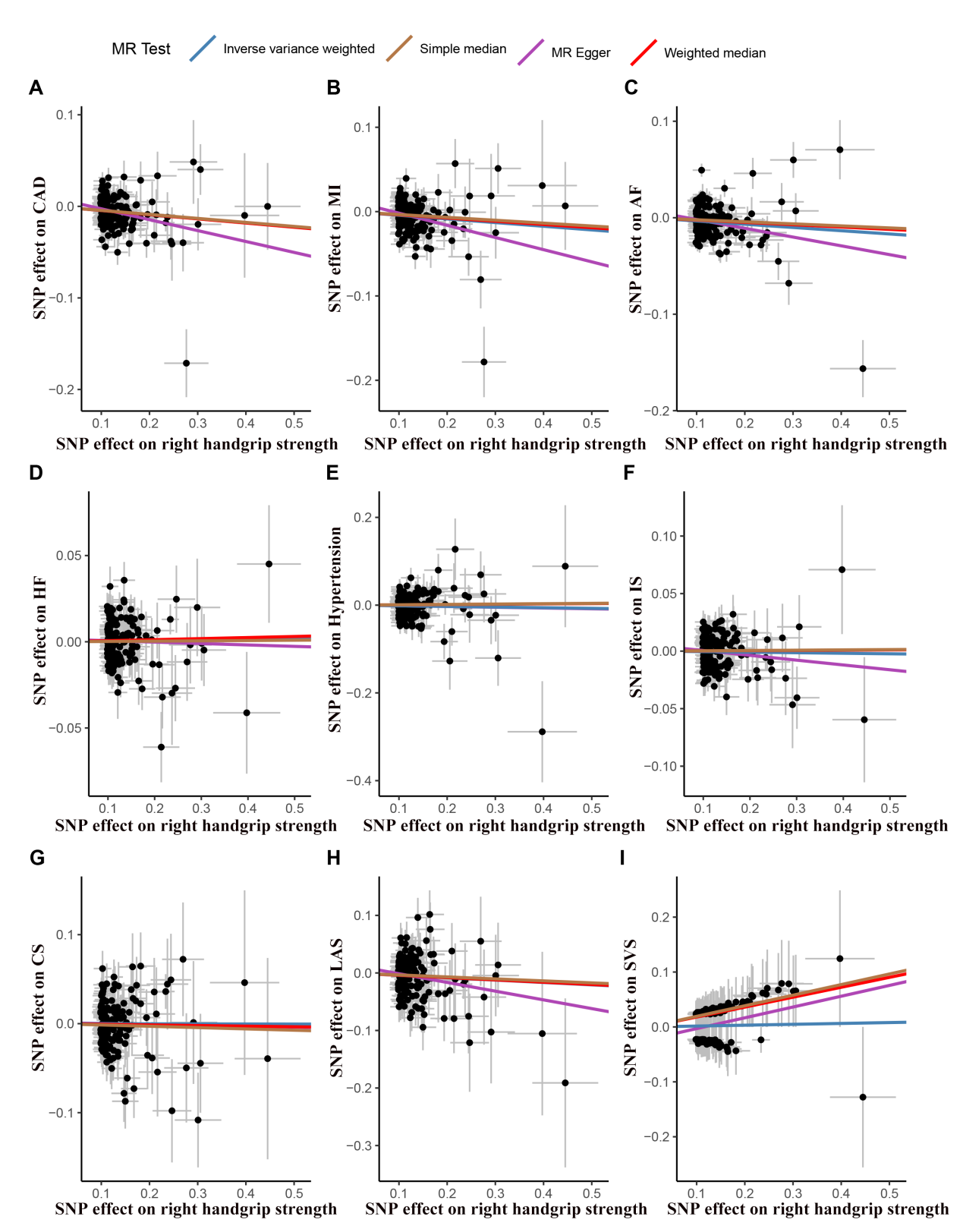


The slope of each line corresponding to estimated Mendelian Randomization (MR) effect per method. Circles indicate marginal genetic associations of each variant with right handgrip strength and the risk of outcomes. Error bars indicate 95% CIs. CAD, coronary artery disease.MI, myocardial infarction.AF, atrial fibrillation.HF, heart failure.IS, ischemic stroke.CS, cardioembolic stroke.LAS, large artery stroke.SVS, small vessel stroke.

# **Supplemental Figure 9.**A scatter plot for the causal association of right handgrip strength with outcomes after removing the SNPs associated with confounders or CVDs.


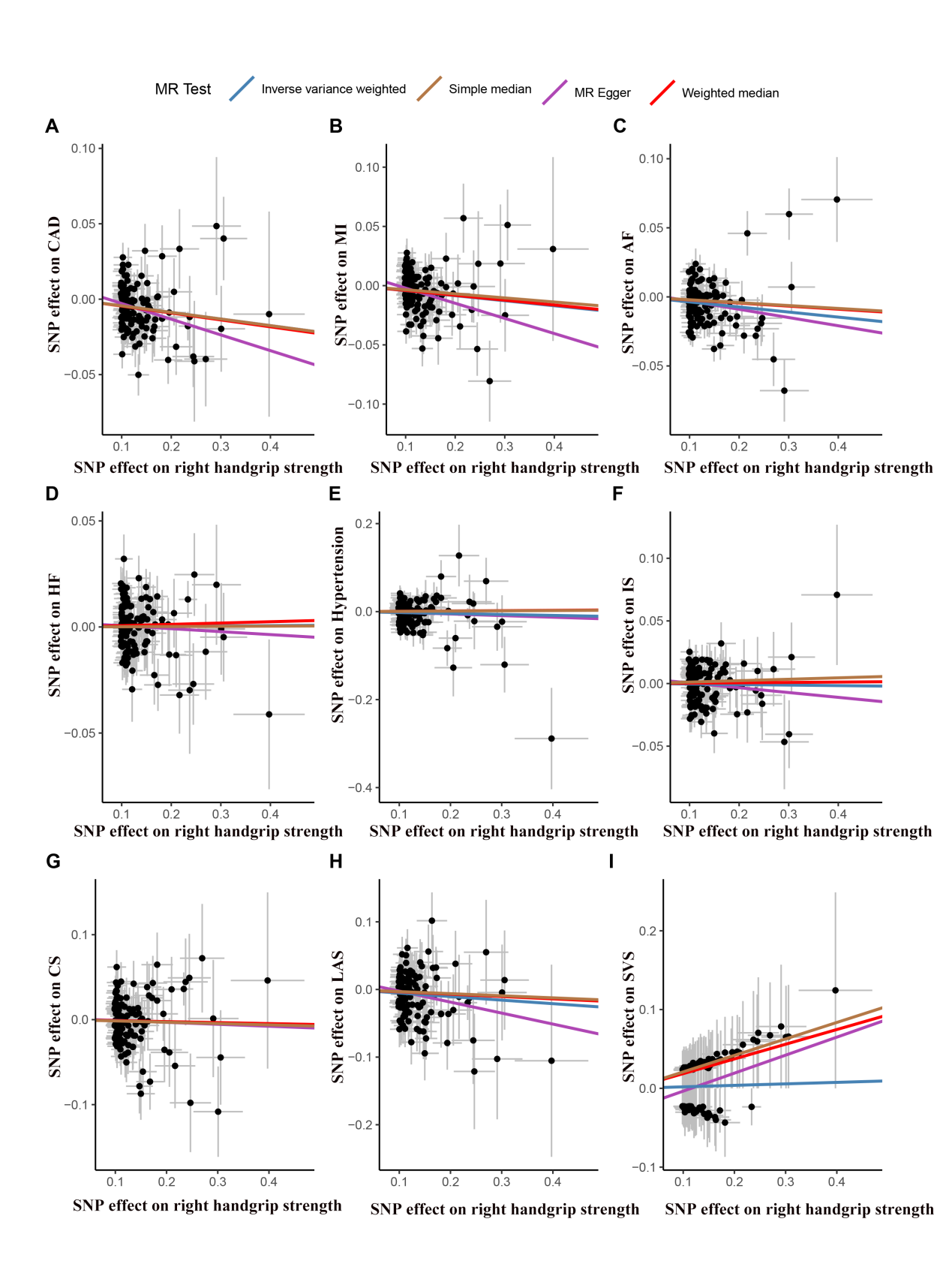


The slope of each line corresponding to estimated Mendelian Randomization (MR) effect per method. Circles represent marginal genetic associations of each variant with right handgrip strength and the risk of outcomes after removing the SNPs associated with confounders or CVDs. Error bars indicate 95% CIs. CADs, coronary artery disease.MI, myocardial infarction.AF, atrial fibrillation.HF, heart failure.IS, ischemic stroke.CS, cardioembolic stroke.LAS, large artery stroke.SVS, small vessel stroke.

# Supplemental Figure 10.A scatter plot for the causal association of left handgrip strength with outcomes.


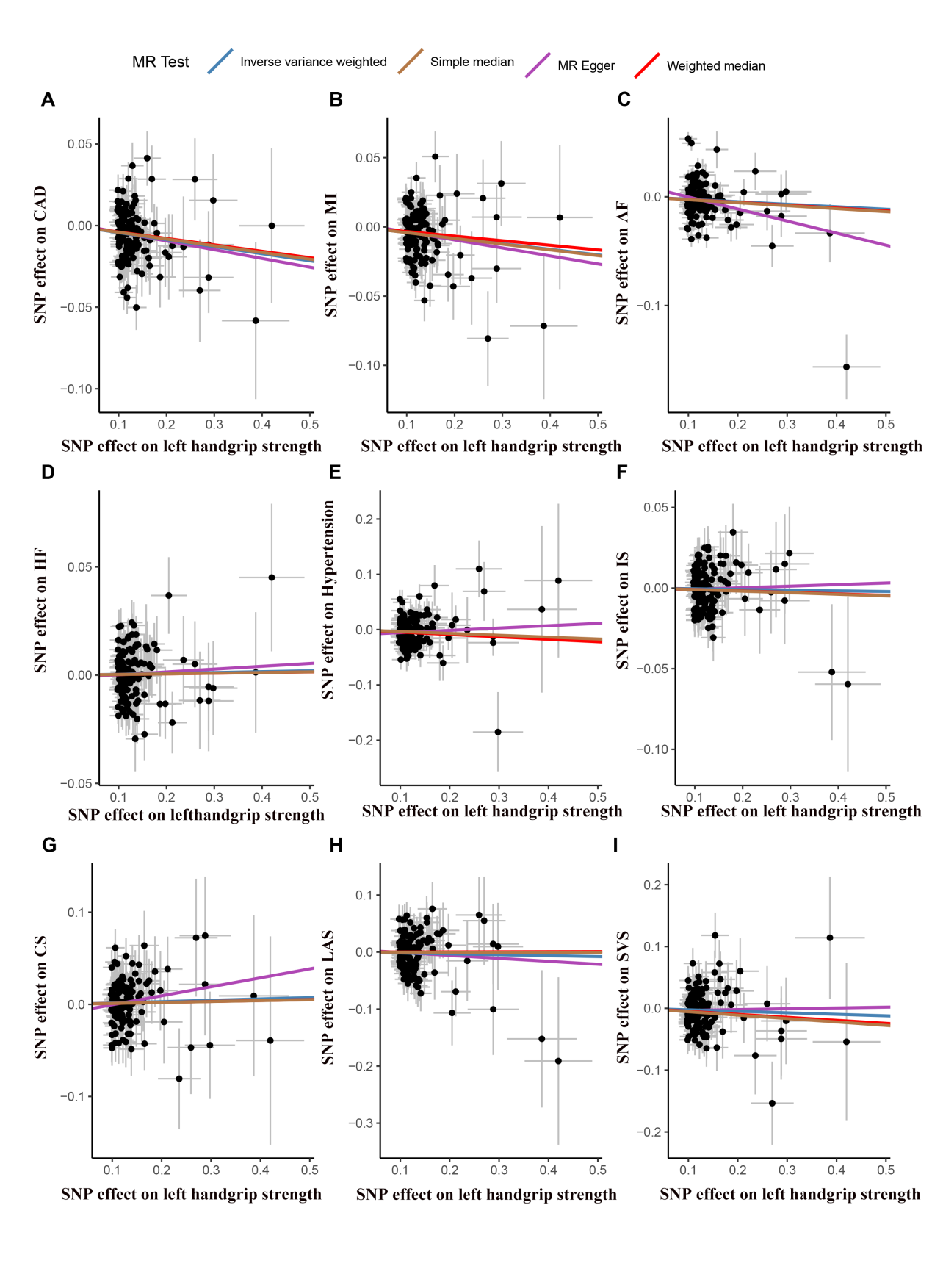


The slope of each colored line corresponding to estimated Mendelian Randomization (MR) effect per method. Circles represent marginal genetic associations of each variant with left handgrip strength and the risk of outcomes. Error bars indicate 95% CIs. CAD, coronary artery disease.MI, myocardial infarction.AF, atrial fibrillation.HF, heart failure.IS, ischemic stroke.CS, cardioembolic stroke.LAS, large artery stroke.SVS, small vessel stroke.

#
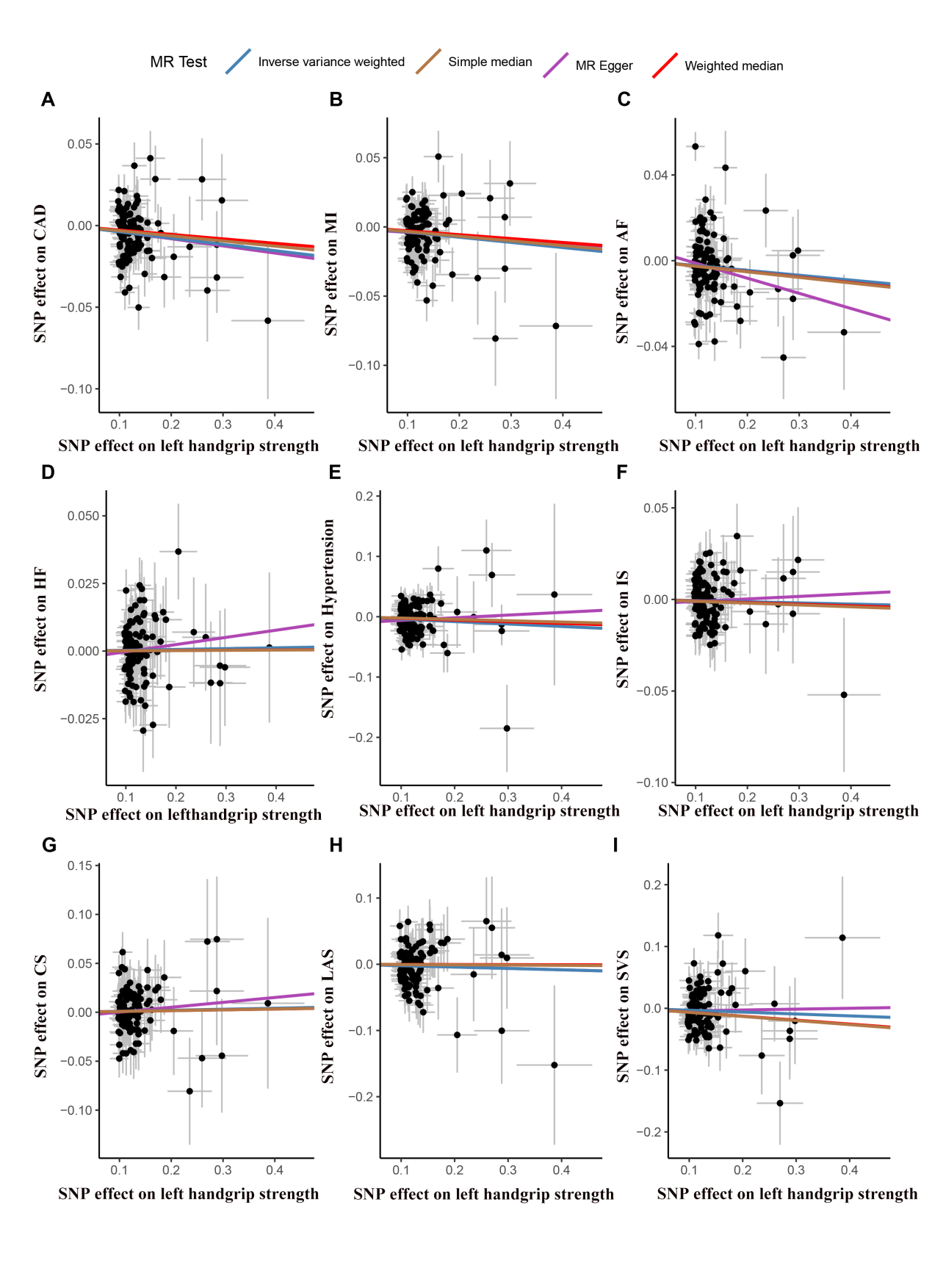
Supplemental Figure 11.A scatter plot for the causal association of right handgrip strength with outcomes after removing the SNPs associated with confounders or CVDs.

The slope of each colored line corresponding to estimated Mendelian Randomization (MR) effect per method. Circles represent marginal genetic associations of each variant with left handgrip strength and the risk of outcomes after removing the SNPs associated with confounders or CVDs. Error bars indicate 95% CIs. CAD, coronary artery disease.MI, myocardial infarction.AF, atrial fibrillation.HF, heart failure.IS, ischemic stroke.CS, cardioembolic stroke.LAS, large artery stroke.SVS, small vessel stroke.
